# Supplementary material for: Microbial drinking water quality deterioration during distribution and household usage, determined together with citizen scientists
Source: PLoS One. 2025 Oct 24;20(10):e0335138. doi: 10.1371/journal.pone.0335138 (PMC12551882; doi:10.1371/journal.pone.0335138)
Supplement: S1 Fig — (DOCX) [file pone.0335138.s001.docx]

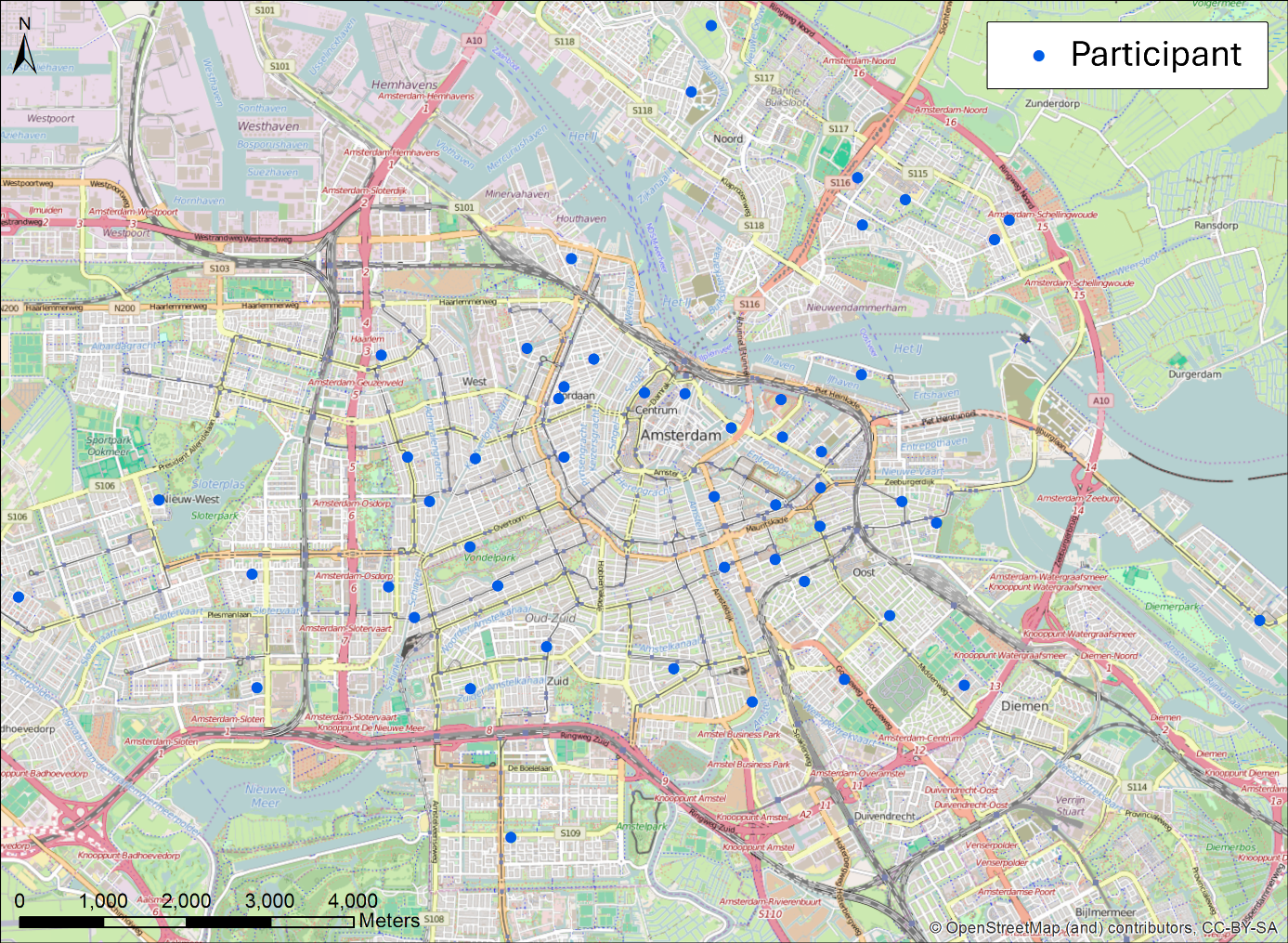


Fig S1. Map of Amsterdam with the locations of the citizen scientists participating in the study marked with blue circles.
