# Supplementary material for: Microbial drinking water quality deterioration during distribution and household usage, determined together with citizen scientists
Source: PLoS One. 2025 Oct 24;20(10):e0335138. doi: 10.1371/journal.pone.0335138 (PMC12551882; doi:10.1371/journal.pone.0335138)
Supplement: S2 Fig — (DOCX) [file pone.0335138.s002.docx]

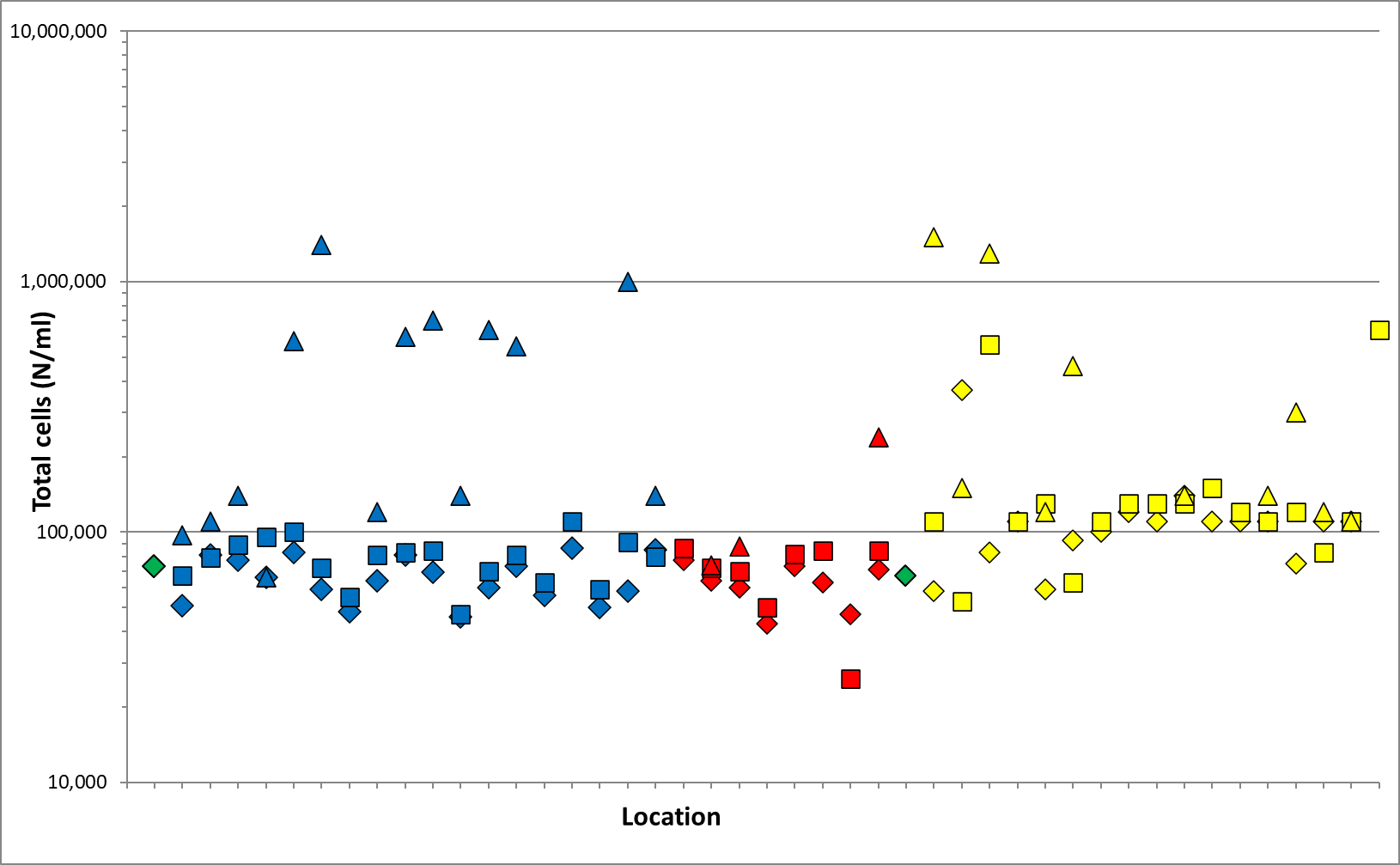


Fig S2. Total cell numbers in drinking water sampled at 43 locations in the distribution system of treatment Plant A (blue symbols), Plant B (yellow symbols), mixed zone of Plant A and B (red symbols) and in treated water sampled at plant A or B (green symbols). Symbols: ◆, Drinking water sampled at the kitchen tap after 5 min flushing (representing drinking water from the distribution system); ■, drinking water sampled directly after opening the kitchen tap after overnight stagnation; ▲, drinking water stored in bottles. The locations are organized per treatment plant from low to high ATP-concentrations in the flushed water samples (see Fig 1).
