# Supplementary material for: Microbial drinking water quality deterioration during distribution and household usage, determined together with citizen scientists
Source: PLoS One. 2025 Oct 24;20(10):e0335138. doi: 10.1371/journal.pone.0335138 (PMC12551882; doi:10.1371/journal.pone.0335138)
Supplement: S1 Table — (DOCX) [file pone.0335138.s004.docx]

Table S1. Number of sequences, OTUs and the Shannon diversity index for each sample.

| Plant | Location | Type | Seqs | OTUs | Shannon |
| --- | --- | --- | --- | --- | --- |
| Plant A | T | Treated | 64058 | 3640 | 6.9 |
| Plant A | 1 | Direct | 62013 | 2846 | 6.0 |
| Plant A | 1 | Flushed | 53047 | 3671 | 7.1 |
| Plant A | 1 | Stored | 51736 | 1121 | 3.8 |
| Plant A | 2 | Direct | 40481 | 2619 | 6.6 |
| Plant A | 2 | Flushed | 32857 | 2789 | 7.0 |
| Plant A | 2 | Stored | 48826 | 499 | 2.7 |
| Plant A | 3 | Direct | 65422 | 3700 | 6.9 |
| Plant A | 3 | Flushed | 65170 | 3699 | 6.6 |
| Plant A | 3 | Stored | 67583 | 1160 | 3.6 |
| Plant A | 4 | Direct | 60552 | 3246 | 6.7 |
| Plant A | 4 | Flushed | 51352 | 3626 | 7.1 |
| Plant A | 4 | Stored | 65175 | 911 | 3.0 |
| Plant A | 5 | Direct | 70794 | 3203 | 6.4 |
| Plant A | 5 | Flushed | 69769 | 3872 | 7.0 |
| Plant A | 5 | Stored | 92430 | 235 | 3.2 |
| Plant A | 6 | Direct | 46344 | 2389 | 5.9 |
| Plant A | 6 | Flushed | 40905 | 3040 | 7.0 |
| Plant A | 6 | Stored | 17797 | 754 | 2.7 |
| Plant A | 7 | Direct | 37305 | 2871 | 7.0 |
| Plant A | 7 | Flushed | 68966 | 4073 | 7.1 |
| Plant A | 7 | Stored | 71587 | 386 | 2.5 |
| Plant A | 8 | Direct | 82961 | 3649 | 6.7 |
| Plant A | 8 | Flushed | 58470 | 3707 | 7.0 |
| Plant A | 9 | Direct | 61179 | 2800 | 5.9 |
| Plant A | 9 | Flushed | 61927 | 4036 | 7.2 |
| Plant A | 9 | Stored | 81776 | 533 | 3.6 |
| Plant A | 10 | Direct | 56226 | 3231 | 6.7 |
| Plant A | 10 | Flushed | 48101 | 3387 | 7.1 |
| Plant A | 10 | Stored | 69057 | 527 | 1.9 |
| Plant A | 11 | Direct | 60152 | 3293 | 6.5 |
| Plant A | 11 | Flushed | 62361 | 4135 | 7.2 |
| Plant A | 12 | Direct | 32428 | 2178 | 6.5 |
| Plant A | 12 | Flushed | 36182 | 2822 | 6.9 |
| Plant A | 13 | Direct | 29972 | 2514 | 6.9 |
| Plant A | 13 | Flushed | 55376 | 3870 | 7.1 |
| Plant A | 13 | Stored | 72158 | 1025 | 3.1 |
| Plant A | 14 | Direct | 66014 | 3628 | 6.9 |
| Plant A | 14 | Flushed | 56442 | 3785 | 7.1 |
| Plant A | 15 | Direct | 61796 | 2918 | 6.4 |
| Plant A | 15 | Flushed | 57743 | 3771 | 7.1 |
| Plant A | 15 | Stored | 86246 | 367 | 1.8 |
| Plant A | 16 | Direct | 52548 | 3033 | 6.3 |
| Plant A | 16 | Flushed | 48801 | 3307 | 7.0 |
| Plant A | 16 | Stored | 58923 | 1646 | 4.6 |
| Plant A | 17 | Direct | 54198 | 3897 | 7.1 |
| Plant A | 17 | Flushed | 55705 | 4218 | 7.3 |
| Plant A | 18 | Direct | 55977 | 2966 | 6.5 |
| Plant A | 18 | Flushed | 56132 | 3734 | 7.1 |
| Plant A | 18 | Stored | 53776 | 1518 | 4.0 |
| Mix | 1 | Direct | 62761 | 3317 | 6.8 |
| Mix | 1 | Flushed | 58672 | 3686 | 7.1 |
| Mix | 2 | Direct | 42868 | 3073 | 6.9 |
| Mix | 2 | Flushed | 39191 | 3168 | 7.1 |
| Mix | 2 | Stored | 1131 | 169 | 4.6 |
| Mix | 3 | Direct | 33872 | 2507 | 6.8 |
| Mix | 3 | Flushed | 28576 | 2625 | 7.0 |
| Mix | 3 | Stored | 60105 | 1325 | 2.6 |
| Plant | Location | Type | Seqs | OTUs | Shannon |
| Mix | 4 | Direct | 41458 | 2871 | 6.8 |
| Mix | 4 | Flushed | 49017 | 3330 | 7.0 |
| Mix | 5 | Direct | 44482 | 2550 | 6.3 |
| Mix | 5 | Flushed | 30025 | 2719 | 7.0 |
| Mix | 5 | Stored | 72442 | 683 | 3.1 |
| Mix | 6 | Direct | 65605 | 2895 | 6.2 |
| Mix | 6 | Flushed | 74760 | 4017 | 7.0 |
| Mix | 6 | Stored | 60631 | 2410 | 5.0 |
| Mix | 7 | Direct | 52901 | 2893 | 6.4 |
| Mix | 7 | Flushed | 48986 | 3310 | 7.0 |
| Mix | 7 | Stored | 92876 | 329 | 3.6 |
| Mix | 8 | Direct | 62954 | 2790 | 6.0 |
| Mix | 8 | Flushed | 78016 | 4668 | 7.2 |
| Mix | 8 | Stored | 66080 | 2568 | 5.6 |
| Plant B | T | Direct | 25060 | 2394 | 6.8 |
| Plant B | 1 | Direct | 65465 | 531 | 2.8 |
| Plant B | 1 | Flushed | 65325 | 4511 | 7.3 |
| Plant B | 1 | Stored | 58879 | 4592 | 7.5 |
| Plant B | 2 | Direct | 77523 | 4101 | 7.2 |
| Plant B | 2 | Flushed | 73950 | 4448 | 7.4 |
| Plant B | 2 | Stored | 79062 | 1312 | 3.0 |
| Plant B | 3 | Direct | 70218 | 3943 | 6.8 |
| Plant B | 3 | Flushed | 41044 | 3347 | 6.9 |
| Plant B | 4 | Direct | 38388 | 2906 | 6.7 |
| Plant B | 4 | Flushed | 42539 | 3138 | 6.8 |
| Plant B | 4 | Stored | 92333 | 2694 | 4.6 |
| Plant B | 5 | Direct | 61640 | 3590 | 6.5 |
| Plant B | 5 | Flushed | 76052 | 4191 | 6.5 |
| Plant B | 6 | Direct | 63833 | 3440 | 6.4 |
| Plant B | 6 | Flushed | 68902 | 3989 | 6.5 |
| Plant B | 7 | Direct | 72126 | 3785 | 6.8 |
| Plant B | 7 | Flushed | 80024 | 4322 | 6.7 |
| Plant B | 7 | Stored | 58360 | 3808 | 7.0 |
| Plant B | 8 | Direct | 70165 | 3847 | 6.9 |
| Plant B | 8 | Flushed | 76271 | 4190 | 7.0 |
| Plant B | 8 | Stored | 76538 | 1370 | 3.5 |
| Plant B | 9 | Direct | 52932 | 3261 | 6.5 |
| Plant B | 9 | Flushed | 39155 | 3068 | 6.5 |
| Plant B | 10 | Flushed | 43863 | 3105 | 6.9 |
| Plant B | 10 | Stored | 31705 | 1344 | 4.4 |
| Plant B | 11 | Direct | 33030 | 2494 | 6.7 |
| Plant B | 11 | Flushed | 5413 | 918 | 6.8 |
| Plant B | 11 | Stored | 55890 | 437 | 2.0 |
| Plant B | 12 | Direct | 72628 | 2107 | 4.2 |
| Plant B | 12 | Flushed | 41024 | 3087 | 6.8 |
| Plant B | 12 | Stored | 56262 | 3574 | 6.9 |
| Plant B | 13 | Direct | 61071 | 4260 | 7.4 |
| Plant B | 13 | Flushed | 54948 | 4107 | 7.2 |
| Plant B | 14 | Direct | 60037 | 2636 | 5.2 |
| Plant B | 14 | Flushed | 38265 | 3352 | 7.4 |
| Plant B | 15 | Direct | 44566 | 2767 | 6.2 |
| Plant B | 15 | Flushed | 32413 | 3010 | 7.2 |
| Plant B | 15 | Stored | 40551 | 1943 | 3.9 |
| Plant B | 16 | Direct | 49813 | 3241 | 6.9 |
| Plant B | 16 | Flushed | 55364 | 4076 | 7.4 |
| Plant B | 16 | Stored | 94286 | 2294 | 4.1 |
| Plant B | 17 | Direct | 82316 | 2244 | 5.0 |
| Plant B | 17 | Flushed | 75747 | 3783 | 6.6 |
| Plant B | 17 | Stored | 98652 | 409 | 4.0 |
