# Supplementary material for: Microbial drinking water quality deterioration during distribution and household usage, determined together with citizen scientists
Source: PLoS One. 2025 Oct 24;20(10):e0335138. doi: 10.1371/journal.pone.0335138 (PMC12551882; doi:10.1371/journal.pone.0335138)
Supplement: S2 Table — (DOCX) [file pone.0335138.s005.docx]

Table S2. Taxonomic classification of OTUs with ≥ 10% dominance in the community of flushed, direct or stored drinking water samples as well as their relative abundance in the other water types at the same location. The relative abundance (in %) of the OTUs are heat map colored.

|  |  |  |  |  | Plant A | | |
| --- | --- | --- | --- | --- | --- | --- | --- |
| OTU | Class | Order | Family | Genus | Flushed | Direct | Stored |
|  |  |  |  |  | Location 1 | | |
| 64 | Actinobacteria | Solirubrobacterales | Solirubrobacterales_unclassified | Solirubrobacterales_unclassified | 0.15 | 10.0 | 0.50 |
| 5 | Betaproteobacteria | Burkholderiales | Comamonadaceae | Acidovorax | 0.0075 | 0 | 14.2 |
| 17 | Sphingobacteria | Sphingobacteriales | Chitinophagaceae | Sediminibacterium | 0.0094 | 0.029 | 14.2 |
|  |  |  |  |  | Location 2 | | |
| 5 | Betaproteobacteria | Burkholderiales | Comamonadaceae | Acidovorax | 0.024 | 0.099 | 30.1 |
| 14 | Alphaproteobacteria | Caulobacterales | Caulobacteraceae | Brevundimonas | 0 | 0 | 13.3 |
| 23 | Alphaproteobacteria | Sphingomonadales | Sphingomonadaceae | Sphingopyxis | 0.0091 | 0.2 | 11.5 |
| 6 | Gammaproteobacteria | Pseudomonadales | Moraxellaceae | Acinetobacter | 0.018 | 0.0099 | 10.5 |
|  |  |  |  |  | Location 3 | | |
| 178 | Actinobacteria | Actinomycetales | Dermabacteraceae | Brachybacterium | 10.2 | 0 | 0 |
| 2 | Betaproteobacteria | Burkholderiales | Comamonadaceae | Comamonadaceae_unclassified | 0.023 | 0.18 | 29.2 |
|  |  |  |  |  | Location 4 | | |
| 36 | Gammaproteobacteria | Gammaproteobacteria_unclassified | Gammaproteobacteria_unclassified | Gammaproteobacteria_unclassified | 0.15 | 3.2 | 25.6 |
| 20 | Alphaproteobacteria | Sphingomonadales | Sphingomonadales_unclassified | Sphingomonadales_unclassified | 0.0078 | 0.035 | 21.0 |
| 183 | Gammaproteobacteria | Pseudomonadales | Pseudomonadaceae | Pseudomonas | 0 | 0 | 10.0 |
|  |  |  |  |  | Location 5 | | |
| 58 | Verrucomicrobiae | Verrucomicrobiales | Verrucomicrobiaceae | Prosthecobacter | 0 | 0 | 18.7 |
| 17 | Sphingobacteria | Sphingobacteriales | Chitinophagaceae | Sediminibacterium | 0 | 0.10 | 12.9 |
| 18 | Betaproteobacteria | Burkholderiales | Burkholderiaceae | Limnobacter | 0 | 0 | 12.7 |
| 105 | Sphingobacteria | Sphingobacteriales | Sphingobacteriales_unclassified | Sphingobacteriales_unclassified | 0 | 0.0028 | 11.1 |
|  |  |  |  |  | Location 6 | | |
| 10 | Gammaproteobacteria | Pseudomonadales | Moraxellaceae | Enhydrobacter | 0.39 | 13.4 | 70.7 |
|  |  |  |  |  | Location 7 | | |
| 41 | Gammaproteobacteria | Pseudomonadales | Pseudomonadaceae | Pseudomonas | 0 | 0 | 32.7 |
| 56 | Sphingobacteria | Sphingobacteriales | Chitinophagaceae | Chitinophagaceae_unclassified | 0 | 0 | 25.0 |
|  |  |  |  |  | Location 9 | | |
| 35 | Gammaproteobacteria | Pseudomonadales | Moraxellaceae | Acinetobacter | 0 | 0 | 19.1 |
| 86 | Gammaproteobacteria | Gammaproteobacteria_unclassified | Gammaproteobacteria_unclassified | Gammaproteobacteria_unclassified | 0 | 0 | 18.2 |
|  |  |  |  |  | Location 10 | | |
| 5 | Betaproteobacteria | Burkholderiales | Comamonadaceae | Acidovorax | 0.031 | 0.023 | 56.7 |
| 14 | Alphaproteobacteria | Caulobacterales | Caulobacteraceae | Brevundimonas | 0 | 0 | 14.9 |
| OTU | Class | Order | Family | Genus | Flushed | Direct | Stored |
|  |  |  |  |  | Location 13 | | |
| 5 | Betaproteobacteria | Burkholderiales | Comamonadaceae | Acidovorax | 0.061 | 0.10 | 20.1 |
| 6 | Gammaproteobacteria | Pseudomonadales | Moraxellaceae | Acinetobacter | 0.011 | 0.62 | 19.0 |
| 86 | Gammaproteobacteria | Gammaproteobacteria_unclassified | Gammaproteobacteria_unclassified | Gammaproteobacteria_unclassified | 0 | 0 | 18.2 |
|  |  |  |  |  | Location 15 | | |
| 6 | Gammaproteobacteria | Pseudomonadales | Moraxellaceae | Acinetobacter | 0.0034 | 0.078 | 50.9 |
| 19 | Gammaproteobacteria | Pseudomonadales | Pseudomonadaceae | Pseudomonas | 0.0035 | 0 | 13.2 |
| 107 | Gammaproteobacteria | Pseudomonadales | Moraxellaceae | Acinetobacter | 0 | 0.024 | 11.8 |
|  |  |  |  |  | Location 16 | | |
| 10 | Gammaproteobacteria | Pseudomonadales | Moraxellaceae | Enhydrobacter | 0.043 | 14.9 | 3.9 |
| 66 | Actinobacteria | Actinomycetales | Geodermatophilaceae | Geodermatophilaceae_unclassified | 0 | 0 | 13.5 |
|  |  |  |  |  | Location 18 | | |
| 93 | Alphaproteobacteria | Rhizobiales | Bradyrhizobiaceae | Bradyrhizobiaceae_unclassified | 0 | 0.0036 | 21.1 |
| 34 | Alphaproteobacteria | Sphingomonadales | Sphingomonadaceae | Sphingobium | 0 | 0.0089 | 20.5 |
|  |  |  |  |  |  | | |
|  |  |  |  |  | Mixed | | |
| OTU | Class | Order | Family | Genus | Flushed | Direct | Stored |
|  |  |  |  |  | Location 2 | | |
| 398 | Bacilli | Bacillales | Bacillales_unclassified | Bacillales_unclassified | 0.15 | 0.056 | 17.4 |
|  |  |  |  |  | Location 3 | | |
| 21 | Alphaproteobacteria | Rhizobiales | Methylobacteriaceae | Methylobacterium | 0 | 0 | 67.3 |
|  |  |  |  |  | Location 5 | | |
| 81 | Betaproteobacteria | Burkholderiales | Burkholderiales_unclassified | Burkholderiales_unclassified | 2.0 | 10.7 | 0.0041 |
| 48 | Alphaproteobacteria | Sphingomonadales | Sphingomonadaceae | Sphingomonadaceae_unclassified | 0 | 0.0067 | 21.7 |
| 20 | Alphaproteobacteria | Sphingomonadales | Sphingomonadales_unclassified | Sphingomonadales_unclassified | 0.010 | 0.14 | 19.3 |
| 17 | Sphingobacteria | Sphingobacteriales | Chitinophagaceae | Sediminibacterium | 0.010 | 0.070 | 12.3 |
|  |  |  |  |  | Location 6 | | |
| 3 | Gammaproteobacteria | Pseudomonadales | Moraxellaceae | Acinetobacter | 0.0027 | 0 | 27.5 |
|  |  |  |  |  | Location 7 | | |
| 18 | Betaproteobacteria | Burkholderiales | Burkholderiaceae | Limnobacter | 0 | 0 | 14.6 |
| 7 | Betaproteobacteria | Burkholderiales | Comamonadaceae | Comamonadaceae_unclassified | 0.51 | 0.39 | 13.6 |
| 126 | Gammaproteobacteria | Xanthomonadales | Xanthomonadales_unclassified | Xanthomonadales_unclassified | 0 | 0 | 10.2 |
|  |  |  |  |  | Location 8 | | |
| 12 | Bacteria_unclassified | Bacteria_unclassified | Bacteria_unclassified | Bacteria_unclassified | 0.36 | 12.8 | 8.2 |
|  |  |  |  |  |  |  |  |
|  |  |  |  |  |  |  |  |
|  |  |  |  |  | Plant B | | |
| OTU | Class | Order | Family | Genus | Flushed | Direct | Stored |
|  |  |  |  |  | Location 1 | | |
| 33 | Betaproteobacteria | Burkholderiales | Burkholderiales_incertae_sedis | Aquabacterium | 0 | 0.011 | 26.6 |
| 17 | Sphingobacteria | Sphingobacteriales | Chitinophagaceae | Sediminibacterium | 0.022 | 0.021 | 13.7 |
|  |  |  |  |  | Location 2 | | |
| 3 | Gammaproteobacteria | Pseudomonadales | Moraxellaceae | Acinetobacter | 0.0027 | 0.0026 | 19.4 |
| 35 | Gammaproteobacteria | Pseudomonadales | Moraxellaceae | Acinetobacter | 0 | 0 | 15.4 |
| 106 | Gammaproteobacteria | Pseudomonadales | Moraxellaceae | Acinetobacter | 0 | 0 | 13.7 |
| 2 | Betaproteobacteria | Burkholderiales | Comamonadaceae | Comamonadaceae_unclassified | 0.22 | 0.16 | 10.9 |
|  |  |  |  |  | Location 4 | | |
| 1 | Betaproteobacteria | Burkholderiales | Burkholderiaceae | Polynucleobacter | 13.6 | 11.3 | 2.6 |
| 31 | Alphaproteobacteria | Sphingomonadales | Sphingomonadaceae | Sphingomonadaceae_unclassified | 0 | 0 | 18.7 |
| 14 | Alphaproteobacteria | Caulobacterales | Caulobacteraceae | Brevundimonas | 0 | 0 | 11.4 |
| 49 | Alphaproteobacteria | Sphingomonadales | Sphingomonadaceae | Sphingomonas | 0 | 0 | 10.8 |
|  |  |  |  |  | Location 7 | | |
| 1 | Betaproteobacteria | Burkholderiales | Burkholderiaceae | Polynucleobacter | 15.0 | 8.7 | 9.2 |
|  |  |  |  |  | Location 8 | | |
| 26 | Betaproteobacteria | Burkholderiales | Burkholderiales_incertae_sedis | Sphaerotilus | 0.038 | 0.019 | 22.7 |
| 18 | Betaproteobacteria | Burkholderiales | Burkholderiaceae | Limnobacter | 0 | 0 | 20.7 |
| 32 | Betaproteobacteria | Betaproteobacteria_unclassified | Betaproteobacteria_unclassified | Betaproteobacteria_unclassified | 0.26 | 0.18 | 17.4 |
|  |  |  |  |  | Location 10 | | |
| 77 | Alphaproteobacteria | Sphingomonadales | Sphingomonadaceae | Sphingomonadaceae_unclassified | NM | 0.34 | 21.2 |
| 66 | Actinobacteria | Actinomycetales | Geodermatophilaceae | Geodermatophilaceae_unclassified | NM | 0.046 | 15.7 |
|  |  |  |  |  | Location 11 | | |
| 19 | Gammaproteobacteria | Pseudomonadales | Pseudomonadaceae | Pseudomonas | 0 | 0.030 | 58.8 |
| 31 | Alphaproteobacteria | Sphingomonadales | Sphingomonadaceae | Sphingomonadaceae_unclassified | 0 | 0 | 10.1 |
|  |  |  |  |  | Location 12 | | |
| 27 | Alphaproteobacteria | Rhizobiales | Bradyrhizobiaceae | Bradyrhizobiaceae_unclassified | 0.85 | 26.2 | 0.76 |
|  |  |  |  |  | Location 15 | | |
| 22 | Betaproteobacteria | Betaproteobacteria_unclassified | Betaproteobacteria_unclassified | Betaproteobacteria_unclassified | 1.0 | 10.1 | 1.3 |
| 10 | Gammaproteobacteria | Pseudomonadales | Moraxellaceae | Enhydrobacter | 0.068 | 1.5 | 54.8 |
|  |  |  |  |  | Location 16 | | |
| 37 | Gammaproteobacteria | Chromatiales | Chromatiaceae | Rheinheimera | 0.043 | 0.020 | 21.8 |
| 14 | Alphaproteobacteria | Caulobacterales | Caulobacteraceae | Brevundimonas | 0 | 0 | 17.4 |
|  |  |  |  |  | Location 17 | | |
| 8 | Betaproteobacteria | Burkholderiales | Burkholderiales_incertae_sedis | Aquabacterium | 0.077 | 13.5 | 2.0 |
