# Supplementary material for: Microbial drinking water quality deterioration during distribution and household usage, determined together with citizen scientists
Source: PLoS One. 2025 Oct 24;20(10):e0335138. doi: 10.1371/journal.pone.0335138 (PMC12551882; doi:10.1371/journal.pone.0335138)
Supplement: Attachment — Submitted filename: Rebuttal.docx [file pone.0335138.s007.docx]

*Reviewer #1: The paper addresses an important and timely topic: the vulnerability of non-chlorinated drinking water to microbial deterioration during distribution, stagnation, and storage, using a citizen science approach. This is highly relevant for public health monitoring and community engagement, particularly in regions like the Netherlands where drinking water is distributed without residual disinfectants. However, the manuscript, while methodologically sound in many respects, suffers from a few limitations in clarity and structure. These include inconsistencies in phrasing, unclear sample classification, and a lack of discussion around potential variability introduced by citizen-led procedures. The limitations of dipslide-based microbial counts, the need for standardization in sampling and incubation, and clarification of the analytical pipeline, should be addressed to strengthen the scientific rigor and readability of the manuscript. The conclusions should be more cautious given the exploratory nature and dataset limitations.*

We thank the reviewer for the extensive review of our manuscript and the comments and suggestions made, which helped us to improve the manuscript, especially on the statistical analysis part. The points raised above are also given in the specific comments of the reviewer and below is our point-to-point response to each of these comments. Please note that the reference to line numbers in the comments of the reviewer refers to the original submission, whereas the reference to line numbers in our reply refers to the revised manuscript with track changes.

*Abstract*

1. *Lines 15–17: The opening sentence is overly long and could be made more digestible.*

The sentence has been shortened (Lines 15-16).

2. *Lines 20–21: The phrase “prokaryotic cell counts using flow cytometry, ATP concentrations and the prokaryotic community composition was determined” contains a subject-verb agreement error and could be clearer.*

The subject-verb agreement was corrected, and the sentence was made clearer (Lines 20-22).

3. *Line 23: Replace "demonstrated" with "showed" or "confirmed" for smoother flow.*

We replaced demonstrated with showed.

4. *Line25: "could result in an increase or decrease of microbial biomass parameters" change to “could result in fluctuations in microbial biomass parameters”*

Changed accordingly.

5. *Line 27 : “dramatically increased microbial biomass” change to “substantially increased microbial biomass”*

Changed accordingly, also at other places in the manuscript.

6. *Line 30: “Supposedly mixed zones” sounds speculative. Clarify whether these zones are known or hypothesized.*

We understand the confusion. Supposedly was used, because the drinking water companies sometimes do not exactly know where drinking water types are mixed in their distribution system. However, you can also read it as being speculative. For clearance, we removed the word supposedly from the sentence.

7. *Line 32–33: This sentence feels too loaded and would benefit from being split.*

This sentence has been split and rephrased, also in relation to a comment made by Reviewer 2 (Lines 31-34).

8. *Line 34: “drinking water is a product prone to microbial deterioration during storage” change to “stored drinking water is highly susceptible to microbial deterioration”*

Changed accordingly.

*Introduction*

9. *Lines 37–38: “In contrast to many other countries, drinking water in the Netherlands is distributed without a disinfectant residual.”; Lines 47–48: “Due to the high quality of drinking water and absence of odour/taste issues related to a disinfectant residual…”. These two statements could be merged or simplified to reduce redundancy.*

We have rephrased this part to eliminate the redundancy (lines 40-41).

10. *Line 15: “premise plumbing systems”; Line 38 and onward: “premises plumbing system” (used multiple times). Standardize to either "premise plumbing system" or "premises plumbing system" throughout for consistency.*

Changed consistently in premises plumbing system(s).

11. *Lines 48–52: “Due to the high quality of drinking water and absence of odour/taste issues related to a disinfectant residual, it is common practice in the Netherlands that consumers refill their containers (i.e. bottles, water cookers, coffee reservoirs, etcetera) with drinking water from the tap. This offers both environmental and costs advantages…” This sentence could be broken into smaller units to improve readability and highlight the key message.*

Sentence has been shortened (Lines 52-54).

12. *Line 47–48: “Due to the high quality of drinking water and absence of odour/taste issues related to a disinfectant residual…”. Consider reframing this as an observed behavior rather than a causal inference unless backed by behavioral data.*

We have rephrased this part in relation to comment 9, which also resolves this comment.

13. *Lines 73–79: “Drinking water quality in the Netherlands is routinely monitored… heterotrophic plate counts at 22°C (HPC), Aeromonas plate counts and Legionella plate counts… ATP, AOC, biomass production potential, cell counts…”. This technical detail could be summarized more generally here and elaborated in the Methods section*.

This part does not describe the analysis we did in our study, but it shows what parameters drinking water companies in The Netherlands routinely monitor to determine the microbiological drinking water quality. Since not all these parameters were measured in our study, it does not fit into the Materials and Methods section that only describes the methods used in our study. As such, we have kept this sentence as it was.

14. *Line 54: “active microbial biomass”; Line 57: “active biomass (ATP)”; Line 67: “active biomass (ATP)”. Use consistent terminology and clarify what "active biomass" refers to throughout.*

We have defined that ATP is used as a measure for active biomass on lines 60-61. We also used active biomass as the consistent terminology throughout the revised manuscript.

15. *Lines 80–81:“This implies that the effect of drinking water storage on the microbial water quality might differ between drinking water treatment plants.” Consider citing specific studies or evidence to substantiate this hypothesis.*

This hypothesis is derived from the observation that microbiological water quality parameters, biological stability parameters and bacterial community composition differs between production locations, which we think is clearly described in the preceding sentence. We feel that the word ‘This’ in the sentence clearly points to these differences described directly before this sentence. As such, we have kept this sentence as it is.

16. L*ines 89–94:“…a ‘new dawn’ of citizen science… One of the issues involving citizen scientists… is whether they can provide reliable microbial sampling…”. More explanation on why citizen scientists are beneficial (e.g., scalability, trust-building, spatial coverage) would strengthen this section*.

It is beyond the scope of our manuscript to write a (show) review on potential and limitations of citizen science in the Introduction section, but such aspects are described in the references provided. Therefore, we added that these aspects can be found in the references provided (Lines 97-98).

17. *Between lines 44–47 and 48–54:There is a jump from technical and aesthetic complaints to consumer storage practices. Add a transition to clarify how consumer behavior is a logical next step in the narrative.*

See our reply to comment 9 and 12.

Materials and Method

18. *Lines 108–114 can be improved by avoiding repetition of the full treatment chains. Instead, emphasize the key differences between the two plants: Plant A supplies the western part of Amsterdam and includes an additional dune infiltration step, while Plant B supplies the eastern part and omits this step. The dune infiltration in Plant A may enhance microbial stability and influence the bacterial community composition of the treated water.*

Changed accordingly.

19. *Line 120: The study relies on participants sampling water after overnight stagnation, but it’s not clear if standardized instructions were given (e.g., stagnation duration, handling). Variability here may affect microbial load and comparability.*

Participants watched an instruction video on how samples have to be taken during a meeting with them and this video remained accessible through a website that was active during the runtime of the project. We added this information on lines 132-135.

20. *Lines 125-126: The phrase "the other 13 citizen scientists choose not to sample drinking water" is a bit misleading. It sounds like they didn’t participate at all. The sentence "non-drinking water samples were omitted" is vague. What does "non-drinking water" mean in this context? The logic flow between who did what and what was analyzed could be more explicit.*

We have changed the text to make this aspect more explicit on lines 143-145.

21. *Lines 137–141: Using citizen scientists to conduct bacterial and fungal plate counts on dipslides is practical but lacks precision and introduces variability. Room temperature incubation is not standardized, and subjective CFU counting by untrained users is prone to bias. In addition, the text lacks info on whether participants were trained, how they handled aseptic techniques, or if contamination risks were managed.*

One of the aims was to determine whether citizen scientists can be used to reliable sample drinking water for microbiology. The dip slides used were chosen because they have been developed for testing by non-trained personnel and the manual instructions of the methods have been followed by the participants (as stated on line 159), limiting variability and bias. The limited variability and bias can be seen by the results from the drinking water samples, which showed no aberrant colony counts, ATP concentrations, cell counts or community composition between samples taken by the different citizen scientists nor with the samples that were taken and analyzed by trained personnel (as described in the Results section). Furthermore, keeping citizen scientists motivated requires more than just sampling drinking water. Therefore, the cultivation on dip slides was a crucial part for the citizen scientist’s involvement. One of the remarkable outcomes is that our study indicates that, when instructed properly, citizen scientists can reliably sample and analyze drinking water (as we concluded on lines 688-690).

22. *Lines 165–179: are thorough, but it would be good to specify: final read depth per sample, percentage of reads removed during filtering steps, normalization method before downstream analysis.*

The final read depth has been added (Lines 207-208). The percentage of reads removed during filtering steps has not been documented and cannot be given. The normalization method has been described on lines 213-219.

23. *The PERMANOVA assumptions (e.g., homogeneity of dispersion) are not discussed. State whether tests like PERMDISP were conducted to verify validity of PERMANOVA results.*

We thank the reviewer for pointing this out and based on this comment we have performed a PERMDISP analysis. The results of the PERMDISP have been added to the results section where the PERMANOVA results are presented (lines 387-390, 402-403 and 415-417).

Results

24. *Lines 199 & 202: The use of “< 1.0 ng ATP/l” without defining the detection limit or measurement threshold is unclear. Explicitly state the detection limit of the ATP assay and whether "< 1.0" reflects below detection or censoring.*

The detection limit of the ATP assay was added to line 167.

25. *Lines 197–198 and 200–201: The repeated phrasing “flushed drinking water samples taken by the citizen scientists whose houses were supplied with drinking water from the distribution system of plant A/B” is overly long and redundant. Replace with concise alternatives such as “citizen samples from Plant A’s distribution zone.”*

Changed accordingly

26. *Although averages are given for ATP levels from Plant A (1.3 ± 0.8 ng/l) and Plant B (2.1 ± 1.0 ng/l), no statistical test is presented to assess whether the difference is significant. Include a statistical comparison (e.g., t-test or Mann-Whitney U) to support any claims of difference between distribution zones.*

We believe that the reviewer refers here to the section with subheading ‘Influence of distribution, overnight stagnation and storage of drinking water’. We refer to Table 1 in this section to show differences in ATP concentrations between samples taken at the treatment plant, directly and after flushing at the tap, and after storage. However, Table 1 also contains information on the differences between Plant A and Plant B. However, that comparison is described in the section with subheading “Influence of treatment plant”. There, we also presented the results from the statistical test on differences in ATP-concentrations between Plant A samples, Plant B samples and samples that received either Plant A or Plant B. Since three groups are compared an ANOVA test with Bonferroni posthoc test was done. We refer the reviewer to lines 488-492 where this part is described.

27. *The section is purely descriptive; it doesn’t comment on what the difference in ATP levels may imply in terms of microbial stability, water age, or plant performance. Briefly interpret the implications—e.g., “This may suggest higher biological stability of water from Plant A.”*

In our study, ATP was only determined as a measure of active biomass. Our previous research showed that ATP is not a reliable indicator for (micro)biological stability, plant performance or water age (van der Wielen & van der Kooij, 2010, Water Res 2010 Vol. 44 Issue 17 Pages 4860-4867 and van der Wielen et al. 2023, Science of The Total Environment 2023 Vol. 871 Pages 161930). However, ATP can be used as an indication for regrowth, maintenance or death. This was already partly included in the original manuscript where we stated that: “At 19 of these 25 locations the ATP increased with more than 20%, indicating microbial growth in the premises plumbing system during overnight stagnation at these 19 locations.”, and “Practically all locations showed more than 20% higher ATP concentrations or cell numbers in stored drinking water than in flushed drinking water samples from the tap, demonstrating microbial growth occurs during drinking water storage in different containers.” On other locations, we included additional text to shortly describe the implications of ATP-changes (Lines 276-277, 290-293, 487-488).

28. *Line 196: The opening sentence states the ATP concentration “in the treated water” but doesn’t clarify whether these values were measured directly at the treatment plants, when, or under what conditions. Clarify if these measurements were made at the plant outlets and under comparable temporal conditions (e.g., also in June 2016).*

This information is provided in the Materials and Methods section on lines 147-148.

29. *Lines 311–320: The observation of dramatic shifts in bacterial community composition in stored water is well reported. However, the term “dramatic” (line 315) is qualitative and subjective. Use more specific metrics (e.g., % shift in community dissimilarity, diversity indices) to support this claim.*

A percentage shift in community composition is difficult to present here as the percentage shift is not the same for all samples and differs quite a bit between each location where stored drinking water was compared with drinking water from the tap. Therefore, to be more neutral, we omitted the term dramatic in this sentence.

30. *Line 312–314: The association between decreased unclassified taxa and increased relative abundance of Proteobacteria and Actinobacteria is informative, but the biological implications of these shifts are not discussed. Add interpretation: Do these taxa reflect regrowth of environmental bacteria, potential health risks, or biofilm formers?*

The phylum Proteobacteria or the phylum Actinobacteria contain species that differ in their metabolic capacities. For instance, the phylum Proteobacteria contain species that can oxidize ammonia, reduce sulfate, aerobically or anaerobically oxidize organic compounds, can be pathogenic, non-pathogenic or even beneficial. Consequently, reliable assigning function to a taxonomic phylum is too speculative in our opinion. We also refer to our reply to comment 37.

31. *Lines 327–338 : The use of PCoA and Bray-Curtis dissimilarity is methodologically appropriate. However, the visual summary ("scattered across the PCoA plot") in line 334 should be quantified, e.g., via centroid distances or dispersion measures.*

The centroid distances have been determined and confirmed that the stored drinking water samples scatter across the PCoA plot compared to the drinking water samples from the tap. We added the average centroid distances to the text on lines 392-395.

32. *Line 336–338: The claim that changes in community composition are “specific for each household” is plausible, but should be supported by inter-household variance measures or discussion of household-specific factors (e.g., container type, storage time, temperature).*

As stated in the manuscript, our data show that the community composition shift in stored drinking water is specific for each household. We agree with the reviewer that it is important to study the factors responsible for the community shift during drinking water storage. However, that objective is beyond the aim of our study, where we first wanted to investigate whether drinking water storage affects community composition. Consequently, our experimental setup cannot be used to identify possible factors responsible for the observed community change, as household factors and how these differ between locations were not registered. Now, that we identified storage to affect the community composition, additional studies that include these aspects as well as laboratory-based studies that can investigate the effect of each factor are needed to give more insight into this aspect.

33. *Lines 339–357 : The degree of change due to stagnation vs. distribution is stated, but the magnitude of community dissimilarity between these steps (e.g., PERMANOVA R² values) is not provided. Include statistical effect sizes to support claims of biological relevance.*

We respectfully disagree with the reviewer on this comment. First, as described in Materials and Methods we used the PRIMER-e software for statistical analysis of the beta-diversity. The PERMANOVA manual of PRIMER-e ([PERMANOVA+ for PRIMER:... | PRIMER-e Learning Hub](https://learninghub.primer-e.com/books/permanova-for-primer-guide-to-software-and-statistical-methods)) states: “In summary, while ANOSIM’s R can be interpreted directly as a measure of the size of the between-group differences, PERMANOVA’s pseudo-F (or pseudo-t) cannot necessarily be interpreted in this way. … In addition, in PERMANOVA it is the P-values which should be used as a measure of strength of evidence with respect to any particular null hypothesis.” As a result, the PRIMER-e software does not calculate these R-values and because of this statement in the manual we are reluctant to use R values from PERMANOVA. Second, the null hypotheses (statistically) tested in our study were: (i) overnight stagnation and/or storage of drinking water does not impact the community composition and (ii) drinking water produced from plant A has the same community composition as drinking water produced from plant B. These hypotheses were tested with PERMANOVA as mentioned in the PRIMER-e manual and in both cases the obtained p-values showed that we must reject these null hypotheses, and which is, thus, independent of the magnitude of community dissimilarity. Third, R-values do not necessarily show biological relevance, as in any correlation analysis relations can be spurious. To demonstrate biological relevance, hypothesis driven laboratory research is required, but that is beyond the goal of our study.

34. *Lines 345–349: The conclusion that community composition remained stable during distribution is valid but should be qualified by the limitations of 16S rRNA profiling (e.g., resolution to genus/species level, potential under-detection of low-abundance shifts).*

We agree with the reviewer that we have not shown that the whole community composition remained stable. We only showed that the dominant bacterial OTU community remained stable. We have changed the text at several locations in the text to dominant bacterial OTU community composition.

35. *Line 384–386: Instead of repeating “in containers from different locations,” streamline to “in multiple households.”*

Changed accordingly.

36. *Lines 391–396: The distinction between growth during stagnation and storage is key but could be better highlighted by comparing fold changes or delta relative abundances between time points.*

We provided the actual increase in relative abundance in the text, as we think that this data is most accurate to describe the increase. As the increase differs per OTU, we also included one more reference to Table S2 in the text, as Table S2 gives the actual relative abundance per OTU in flushed, direct and stored drinking water. The reader can see the exact increase or decrease in relative abundance in that Table.

37. *It would help to contextualize the potential ecological or health roles of these dominant OTUs, especially genera like Pseudomonas, Acinetobacter, and Enhydrobacter*.

Many studies have demonstrated that relating function to 16S rRNA gene sequences is unreliable and care should be taken (e.g. Matchado et al. 2024, DOI: 10.1099/mgen.0.001203 and references therein). Therefore, we are reluctant to describe the ecological roles of dominant OTUs and we discuss only briefly some implications in the Discussion section (lines 652-655).

38. *Lines 418–421: The use of statistical testing (ANOVA with Bonferroni) is appropriate and strengthens the conclusion. However, the effect size or p-values should be mentioned explicitly.*

We have included a part in the Materials and Methods on the statistical testing of the ATP-concentrations. First, normality of data was tested with Shapiro-Wilkinson, then homogeneity of variance was tested with Levene’s test. The outcome of these tests showed normality of data and homogeneity of variance. Subsequently, we performed the ANOVA with Bonferroni posthoc. Finally, we included that we used a p-value of 0.05 to reject or accept the null hypothesis. We respectfully disagree with the reviewer about providing actual p-values. We have performed statistics to test whether to accept or reject null hypotheses. Statistical textbooks mention explicitly that before carrying out a statistical analysis to test the null hypothesis, the magnitude of type I error (rejection of hypothesis, p-value) allowed must be determined (e.g. Biometry, The Principles and Practice of Statistics in Biological Research, third edition, Sookal and Rohlf (eds), 1995). We have set this level at p<0.05 as now stated in the Materials and Methods section (lines 169-178). Consequently, in our manuscript we reject the null hypothesis below this p-value and at or above this p-value we accept the null hypothesis, irrespective of the actual p-value.

39. *Line 423: The term “microbial water quality was slightly better” is vague and needs a clearer definition—was this based on ATP only? Or all parameters?*

We thank the reviewer for pointing this out. As already stated in our reply to comment 27 ATP (or cell numbers) cannot be used to determine whether one treatment plant performs better than another. Therefore, we have omitted this sentence from the manuscript.

40. *Lines 426–430: The conclusion that the mixing zone is primarily fed by Plant A is reasonable based on ATP/cell count comparisons, but hydraulic data or modeling would greatly strengthen this inference.*

We agree with the reviewer, but such data is not available.

41. *Line 436–440: Class-level comparisons are mentioned, but the ecological or functional relevance of these shifts is not discussed. For example, is a lower abundance of β-Proteobacteria associated with lower nutrient availability?*

As indicated in our response to comment 30 and 37, relating function to 16S rRNA gene sequences is unreliable and should be prevented.

42. *Line 442–448: The result that the mixing zone samples resemble Plant A samples is convincing, but again, would benefit from quantitative support (e.g., % shared OTUs, similarity indices).*

We agree with the reviewer that based on the data that is currently presented in the manuscript we can convincingly conclude that the mixing zone samples resemble Plant A samples. Additional analyses to prove this aspect are in our opinion not required.

Discussion

43. *Line 455–456: The sentence presents citizen science as a valid approach but lacks a critical view of its limitations (e.g., sampling errors, lack of lab conditions). Include a more balanced assessment by addressing both the benefits and limitations of using citizen scientists for microbiological sampling*.

We included a sentence in the Discussion to point to these potential limitations (lines 525-528), but we would also like to stress that such limitations were not observed in our study.

44. *467–470: Clarify whether variability was due to sampling, analytical technique, or participant handling.*

We refer here to a previous study that does not provide the information requested by the reviewer.

45. *471–473: Add information on whether instruction quality was evaluated (e.g., quiz, feedback), or how citizen scientists' sampling performance was validated pre-study.*

We moved the instruction video part to the Materials and Methods section as requested by reviewer 2 (lines 132-135). Further evaluation was not done and is, therefore, not included in the text in Materials and Methods.

46. *475–477: The claim that no aberrant results were observed is strong but not supported by statistical evidence.*

In this study, only citizen scientists took the direct, flushed and stored drinking water samples. These results were compared with each other and with previous microbiological data from the distribution systems (sampled at other locations). Consequently, a reliable statistical comparison on data collected by citizen scientists and well-trained personnel on the same samples cannot be done. We, therefore, agree with the reviewer that our statement “… clearly showed that no aberrant results …” is too strong and we weakened it to “… indicate that no aberrant results …”.

47. *478–481 The conclusion that citizen scientists can "reliably sample and analyze" is quite broad given that only basic microbiological parameters were assessed. Rephrase to limit the scope of reliability to the tested parameters and context, and emphasize the need for caution in generalizing these findings.*

Changed accordingly (Lines 552-556).

48. *499–501: Improve clarity by first acknowledging that some parameters may appear stable while others show growth, and distinguish between general vs. specific microbial indicators.*

In the sentences directly above these sentences it is written which parameters can be stable during distribution and repeating them again here would be redundant in our opinion. The specific microorganisms have now been specified in the text (Lines 577-578).

49. *508–513: The critique of using ATP and flow cytometry as indicators is valid, but lacks depth regarding what alternatives might be feasible. e.g., qPCR, next-gen sequencing, biosensors*.

In a previous study, it was shown that Aeromonas and heterotrophic plate counts are better indicators for regrowth than ATP or flow cytometry and we included that in the text on lines 586-588. We are not aware of studies that have shown feasibility of qPCR, next-gen sequencing, biosensors as reliable regrowth indicators in distributed drinking water. If, however, the reviewer can provide us with references we are happy to include those studies in the discussion of our manuscript.

50. *533–537: explore potential confounders like plumbing material, usage patterns, or temperature.*

We thank the reviewer for pointing out that other factors could also been the cause for the observed differences. We have included a sentence that these factors might have contributed to the observed differences as well (Lines 618-619).

51. *539–547 Reference studies quantifying copper's antimicrobial effect in situ to support the claim.*

We refer to a review of Vincent et al. (2016) where references to such studies can be find (see line 629).

*Reviewer #2: I have attached an edited Word version of the manuscript with grammatical edits and comments. The authors should review again for grammar and consistency in language. The paper has implications for the field of public health. Public water systems may be in compliance with regard to bacterial contaminants; however, water may become contaminated with bacteria during the distribution system or in containers used to store water. Water containment systems (bottled water) are also at risk of microbial growth. The authors involved citizen scientists in the process of collecting water and conducting some lab analyses, which included bacterial and fungal plate counts.*

We thank the reviewer for his extensive review and the grammatical edits and comments, which helped us to improve our manuscript. The majority of the grammatical edits provided in the attached Word document were accepted. In addition, the revised manuscript was reviewed for grammar and consistency in language and text was corrected when needed. Below, we provide a point-to-point response to the comments made by Reviewer 2. Please note that the reference to line numbers in the comments of the reviewer refers to the original submission, whereas the reference to line numbers in our reply refers to the revised manuscript with track changes.

**Comments made in the Word document**

1. *Is this the main takeaway from this paper?*

The main takeaway from the paper is that drinking water storage has an impact on the microbial water quality. We have changed the order in which the conclusions are presented in the abstract and the last paragraph of the discussion, starting with the most important one.

2. *Explain that ATP is adenosine triphosphate and how these molecules can be used to determine active microorganisms—is there a reference for this?*

This information is now provided on lines 58-61.

3. *Maybe this needs to be described in methods: did you host events, have individual citizen scientists sign up to come in and count CFUs, etc?*

We added this information to the Materials and Methods section (Lines 126-135).

4. *Citizen science really has been a thing over the past couple of decades. I see a paper titled “A New Dawn for Citizen Science” that you do not reference, but you probably should if you are using this phrase.* [*https://www.cell.com/trends/ecology-evolution/abstract/S0169-5347(09)00175-X?cc=y*](https://www.cell.com/trends/ecology-evolution/abstract/S0169-5347(09)00175-X?cc=y)

The reference is included.

5. *There are so many citizen science papers that you could cite here.*

We agree with the reviewer, but we would like to limit the number of references, so we only cite two as examples. To indicate this, we added e.g. before the reference numbers in the text (Lines 97-98).

6. *Where and how did they do these analyses?*

This information is provided on lines 132-135.

7. *Maybe this needs to be described in methods: did you host events, have individual citizen scientists sign up to come in and count CFUs, etc?*

We added this information now on lines 126-135.

8. *I think it should be described in methods how these folks were recruited.*

Our study resulted in two papers. One more on the social aspects of the citizen scientists that was already published in 2018 and one on the microbial drinking water quality presented in this manuscript. In the 2018 paper we explained how these citizen scientists were recruited. We made that now clearer in the Materials and Methods section (Lines 126-127).

9. *What is Figure 1 showing? That levels of bacteria were similar in studies comparing results of citizen scientists with those of technicians.*

Figure 1 shows the ATP concentrations of the drinking water sampled, as stated in the legend of Figure 1. These include samples taken by trained personnel and the citizen scientist, flushed, direct and stored samples taken by the citizen scientists and samples from locations distributed with water from plant A, B or either plant A or B. In the text where the reviewer made the comment, we specifically refer to the ATP-concentrations in the flushed drinking water samples measured by the citizen scientists and that refers to Figure 1 (Lines 234-236).

10. *This should be in the methods in a section called Preparing Citizen Scientists*

We moved this part to the Materials and Methods section (lines 126-135).

11. *Cite the webpage*

The webpage was only active during the project phase and cannot be assessed anymore.

12. *This is a significant result, and belongs in the Results section.*

This information is available in the Results section at lines 244-250.

13. *Or just say “can result in microbial growth in the drinking water obtained at the kitchen tap.*

Changed accordingly.

**Comments made directly to the Editor**

14. *• The authors should consider expanding their methods to clearly define how citizen scientists were recruited and trained, what their role was in the project (for example, in addition to collecting water samples from the tap, it seems that testing water bottles for microbes was the citizen scientists’ idea, so their role was to reliably collect samples, assist in bacterial and fungal plate counts, and pose new questions about sources and locations of drinking water contamination), and when and how they were included in laboratory analyses.*

As indicated in our response to comment 8, information on how citizen scientists were recruited, what their expectations were and how they experienced their contribution and view on drinking water has been described in a separate paper published in 2018 (reference 37 in our manuscript; Brouwer et al. 2018, Water 10(3):284). The other information is now added in the Materials and Methods section (Lines 126-135).

15. *• Are any of the citizen scientists authors?*

It was decided not to include the citizen scientists as co-authors.

16. *• The description of citizen science outcomes in the Discussion should be in Results.*

See our response to comment 12 which is the same comment as this one but which the Reviewer made in the Word document.

17. *• How does this paper build on and/or relate to reference 37? Are there any citizen scientists who overlap between these two endeavors?*

The study we did contained two aspects: one on the social site of including citizen scientists into drinking water quality monitoring and one on the effects of drinking water storage on microbial water quality where sampling was done by citizen scientists, but most of the analyses in a professional laboratory. The social science part is reported in reference 37, whereas the microbial water quality part is described in this manuscript. We made this clearer on two locations in the manuscript (Lines 126-127 and 554-556).

18. *Overall, this is a thorough paper. It is somewhat challenging to determine whether it primarily focuses on citizen science or the contamination of drinking water through distribution systems assisted by citizen scientists. The authors should review each section of the manuscript to highlight which of their approaches was central to the questions addressed in this study.*

We thank the reviewer for this last overall comment. By changing the order of our conclusions in both the Abstract (Lines 31-34) and Conclusions (Lines 675-702), and rephrasing the goals of our study in the Introduction section (Lines 102-105) we have highlighted better the main goal of our study and the most important findings of our study.
