# Supplementary material for: Microbial drinking water quality deterioration during distribution and household usage, determined together with citizen scientists
Source: PLoS One. 2025 Oct 24;20(10):e0335138. doi: 10.1371/journal.pone.0335138 (PMC12551882; doi:10.1371/journal.pone.0335138)
Supplement: Attachment — Submitted filename: Second Review PLOS.docx [file pone.0335138.s008.docx]

Second Review

The edits contribute significantly to the readability and understandability of the manuscript. I am not sure that you can insert references the way you did, adding an "a" rather than re-ordering, but I leave it to the editors to decide how to deal with references.

In clean version, I see some typos and need for grammatical changes:

**Line 119:** Do you mean citizen scientists obtained water samples from these districts? So, they were not just testing their homes, but also collected other samples as well? I am not sure how their homes would be supplied by both districts A and B.

Line 132: you mean “chose” not “choose”.

**Line 150:** houses of citizen scientists

**Line 163-164:** “flushed and direct drinking water and between direct and stored drinking water samples

**Line 174:** Solution BF1 from the kit

**Line 181-182**: perhaps the “Illumina MiSeq 16S Metagenomic sequencing 182 library preparation protocol” should be cited in references rather than as a url in the text of the manuscript.

**Line 189:** same comment for the MiSeq standard operating procedure

**Line 220:** The results being reported here aren’t about the citizen scientists but about their samples. The samples revealed ATP concentrations…that should be the subheading here…” ATP Concentrations from Samples Supplied by Citizen Scientists”. Or even just ….”ATP Concentrations”.

**Line 274**: remove the word “that” to read “indicating active biomass decay”

**Line 300**: Replace “practically all” with “most”

**Line 303**: Replace “had been” with “were”

Line 310-311: Seems odd to describe the shift toward a community of bacteria “capable of growing on an agar medium”. What exactly was the makeup of the bacterial community that was growing on an agar medium? Did the medium select for a particular type? If so, state that.

**Line 428:** Replace “belonged” with “belong”

**Line 487:** Replace “Proteobaceria” with “Proteobacteria.”

**Line 505:** Replace “unexperienced” with “inexperienced”

**Line 543:** a premise’s….this is a recurring issue…I think it should be the possessive in each case throughout the manuscript.

**Line 550-553:** Rather than say that it would be a misconception to infer from the findings that regrowth of bacteria in distribution systems is negligible…Simply state that “although we did not identify significant regrowth of bacteria in distribution systems, other studies have verified that this can occur”…then go on to reference studies with a different outcome from yours.

**Line 553:** I don’t know what en Legionella means.

**Line 590**: cite “previous studies”
