# Supplementary material for: Microbial drinking water quality deterioration during distribution and household usage, determined together with citizen scientists
Source: PLoS One. 2025 Oct 24;20(10):e0335138. doi: 10.1371/journal.pone.0335138 (PMC12551882; doi:10.1371/journal.pone.0335138)
Supplement: Attachment — Submitted filename: Rebuttal_auresp_2.docx [file pone.0335138.s009.docx]

Dear Editor,

We thank the reviewers for their positive judgement of our revised submission and that they noticed that we addressed most of their prior concerns. From the review reports we understood that only reviewer 2 has made some suggestions from improvements and we thank the reviewer for pointing out these improvements. We address these comments below. Please note that the reference to line numbers in the comments of the reviewer refers to the original submission, whereas the reference to line numbers in our reply refers to the revised manuscript with track changes.

*The edits contribute significantly to the readability and understandability of the manuscript. I am not sure that you can insert references the way you did, adding an "a" rather than re-ordering, but I leave it to the editors to decide how to deal with references.*

We have added an ‘a’ rather than reordering the whole reference list in other publications, although these publications were not published in PLOS ONE. We will wait for the editors how they decide on this.

*In clean version, I see some typos and need for grammatical changes:*

*Line 119: Do you mean citizen scientists obtained water samples from these districts? So, they were not just testing their homes, but also collected other samples as well? I am not sure how their homes would be supplied by both districts A and B.*

The 8 houses where the reviewer refers to received either drinking water from plant A or plant B depending on the time of the day. We have clarified this point on lines 119 to 121.

*Line 132: you mean “chose” not “choose”.*

Changed accordingly.

*Line 150: houses of citizen scientists*

Changed accordingly.

*Line 163-164: “flushed and direct drinking water and between direct and stored drinking water samples*

Changed accordingly.

*Line 174: Solution BF1 from the kit*

Changed accordingly.

*Line 181-182: perhaps the “Illumina MiSeq 16S Metagenomic sequencing 182 library preparation protocol” should be cited in references rather than as a url in the text of the manuscript.*

In our previous publications we have always referred to this document in the manuscript text and not in the reference list, as these documents do not have a specified author. Therefore, we have kept the url in the text here.

*Line 189: same comment for the MiSeq standard operating procedure.*

In our previous publications we have always referred to this document in the manuscript text and not in the reference list, as these documents do not have a specified author. Therefore, we have kept the url in the text here.

*Line 220: The results being reported here aren’t about the citizen scientists but about their samples. The samples revealed ATP concentrations…that should be the subheading here…” ATP Concentrations from Samples Supplied by Citizen Scientists”. Or even just ….”ATP Concentrations”.*

In the first part of the results section (i.e. under the first heading in the results section where the reviewer points to) we want to show the sampling performance of the citizen scientists. This section is not about the ATP concentrations we measured as ATP results are also presented under the other headings in the Results section. Therefore, renaming the heading to ATP-concentrations doesn’t seem to cover the results presented here. Therefore, we renamed the heading: Sampling performance by the citizen scientists (line 220).

*Line 274: remove the word “that” to read “indicating active biomass decay”*

Changed accordingly.

*Line 300: Replace “practically all” with “most”*

Changed accordingly.

*Line 303: Replace “had been” with “were”*

Changed accordingly.

*Line 310-311: Seems odd to describe the shift toward a community of bacteria “capable of growing on an agar medium”. What exactly was the makeup of the bacterial community that was growing on an agar medium? Did the medium select for a particular type? If so, state that.*

As described in Materials and Methods we used agar dip slides that detect bacteria. To be more precise we changed the sentence into ‘This result is an indication that not only the biomass increased during drinking water storage but that the community also shifted towards one harboring more bacteria capable of growing on the agar dip slides for bacteria used in our study’ (lines 308-311).

*Line 428: Replace “belonged” with “belong”*

Changed accordingly.

*Line 487: Replace “Proteobaceria” with “Proteobacteria.”*

Changed accordingly.

*Line 505: Replace “unexperienced” with “inexperienced”*

Changed accordingly.

*Line 543: a premise’s….this is a recurring issue…I think it should be the possessive in each case throughout the manuscript.*

According to the English dictionary premise refers to an assertion or proposition, whereas premises refers to the land and buildings that are part of a property. Since the plumbing systems refers to physical property in a building, we believe premises plumbing is correct English. So, we have kept premises throughout the manuscript.

*Line 550-553: Rather than say that it would be a misconception to infer from the findings that regrowth of bacteria in distribution systems is negligible…Simply state that “although we did not identify significant regrowth of bacteria in distribution systems, other studies have verified that this can occur”…then go on to reference studies with a different outcome from yours.*

We have not reliably determined regrowth in our study, because we already know from previous studies that more parameters need to be determined to identify regrowth. We agree with the reviewer that the word misconception might be too strong and, therefore, we have removed that part from the manuscript (lines 550-551).

*Line 553: I don’t know what en Legionella means.*

We apologize for this. ‘en’ is a Dutch word meaning and. We changed that in the revised manuscript.

*Line 590: cite “previous studies”*

Changed accordingly.
