# Supplementary material for: Microbial drinking water quality deterioration during distribution and household usage, determined together with citizen scientists
Source: PLoS One. 2025 Oct 24;20(10):e0335138. doi: 10.1371/journal.pone.0335138 (PMC12551882; doi:10.1371/journal.pone.0335138)
Supplement: Attachment — Submitted filename: PONE-D-25-20999-reviewer comments-2.docx [file pone.0335138.s006.docx]

PLOS ONE

# Microbial drinking water quality deterioration during distribution and household usage, determined together with citizen scientists.

--Manuscript Draft--

| **Manuscript Number:** | PONE-D-25-20999 |
| --- | --- |
| **Article Type:** | Research Article |
| **Full Title:** | Microbial drinking water quality deterioration during distribution and household usage, determined together with citizen scientists. |
| **Short Title:** | Drinking water quality during transport and storage |
| **Corresponding Author:** | Paul van der Wielen  KWR Water Research Institute: KWR Water BV Nieuwegein, NETHERLANDS, KINGDOM OF THE |
| **Keywords:** | Drinking water; Water quality; microbiology; Citizen scientists; Bacteria; 16S rRNA gene analysis; Bacterial community analysis; premise plumbing system; biomass; microbial deterioration |
| **Abstract:** | During transport and storage of drinking water in the distribution system, premise plumbing systems or containers used by consumers, the microbial water quality might deteriorate. Here, we studied the vulnerability of non-chlorinated drinking water produced by two treatment plants to deterioration, by involving citizen scientists. Citizen scientists in Amsterdam sampled their drinking water directly from their kitchen tap after overnight stagnation, after flushing, and after storage in containers like reusable plastic bottles. Subsequently, prokaryotic cell counts using flow cytometry, ATP concentrations and the prokaryotic community composition were determined in the laboratory. The results demonstrated that citizen scientists were able to reliably sample drinking water. The microbiological parameters measured remained stable during drinking water transport in the distribution system, whereas overnight stagnation in the premise plumbing system could result in an increase or decrease of microbial biomass parameters and slightly alter the prokaryotic composition. Drinking water storage in containers resulted in a dramatic increase in microbial biomass, a decrease in bacterial diversity, and a shift in bacterial community composition. Furthermore, sampled drinking water from the distribution system had a specific community composition related to either plant A or B, which could be used to determine the origin of drinking water sampled from supposedly mixed zones in the distribution system. Overall, we conclude that citizen scientists can reliably sample drinking water for microbial analyses, that ATP and flow cytometry cell counts are poor indicators for microbial regrowth in the distribution system, and that drinking water is a product prone to microbial deterioration during storage. |
| **Order of Authors:** | Paul van der Wielen |
|  | Stijn Brouwer |
|  | Marco Dignum |
|  | Merijn Schriks |
| **Additional Information:** |  |
| **Question** | **Response** |
| **Financial Disclosure**  Enter a financial disclosure statement that describes the sources of funding for the work included in this submission. Review the [submission guidelines](http://journals.plos.org/plosone/s/submission-guidelines#loc-financial-disclosure-statement) for detailed requirements. View published research articles from [*PLOS ONE*](https://journals.plos.org/plosone/) for specific | Yes |

| examples.  This statement is required for submission and **will appear in the published article** if the submission is accepted. Please make sure it is accurate. |  |
| --- | --- |
| **Funded studies**  Enter a statement with the following details:   - Initials of the authors who received each award - Grant numbers awarded to each author - The full name of each funder - URL of each funder website - Did the sponsors or funders play any role in the study design, data collection and analysis, decision to publish, or preparation of the manuscript? |  |
| Did you receive funding for this work? |  |
| Please add funding details.  as follow-up to "**Financial Disclosure**  Enter a financial disclosure statement that describes the sources of funding for the work included in this submission. Review the [submission guidelines](http://journals.plos.org/plosone/s/submission-guidelines#loc-financial-disclosure-statement) for detailed requirements. View published research articles from [*PLOS ONE*](https://journals.plos.org/plosone/) for specific examples.  This statement is required for submission and **will appear in the published article** if the submission is accepted. Please make sure it is accurate. | The research was funded by the joint research program of the ten Dutch drinking water companies and one Flemish drinking water company. |
| **Funded studies**  Enter a statement with the following details:   - Initials of the authors who received each award - Grant numbers awarded to each author - The full name of each funder - URL of each funder website - Did the sponsors or funders play any role in the study design, data collection and analysis, decision to publish, or preparation of the manuscript? |  |
|  |  |

| Did you receive funding for this work?" |  |
| --- | --- |
| Please select the country of your main research funder (please select carefully as in some cases this is used in fee calculation).  as follow-up to "**Financial Disclosure**  Enter a financial disclosure statement that describes the sources of funding for the work included in this submission. Review the [submission guidelines](http://journals.plos.org/plosone/s/submission-guidelines#loc-financial-disclosure-statement) for detailed requirements. View published research articles from [*PLOS ONE*](https://journals.plos.org/plosone/) for specific examples.  This statement is required for submission and **will appear in the published article** if the submission is accepted. Please make sure it is accurate. | NETHERLANDS - NL |
| **Funded studies**  Enter a statement with the following details:   - Initials of the authors who received each award - Grant numbers awarded to each author - The full name of each funder - URL of each funder website - Did the sponsors or funders play any role in the study design, data collection and analysis, decision to publish, or preparation of the manuscript? |  |
| Did you receive funding for this work?" |  |
| **Competing Interests**  Use the instructions below to enter a competing interest statement for this submission. On behalf of all authors, disclose any [competing interests](http://journals.plos.org/plosone/s/competing-interests) that could be perceived to bias this work—acknowledging all financial support and any other relevant financial or non- financial competing interests. | The authors have declared that no competing interests exist. |

| This statement is required for submission and **will appear in the published article** if the submission is accepted. Please make sure it is accurate and that any funding sources listed in your Funding Information later in the submission form are also declared in your Financial Disclosure statement.  View published research articles from  [*PLOS ONE*](http://journals.plos.org/plosone/) for specific examples. |  |
| --- | --- |
| **NO authors have competing interests**  Enter: *The authors have declared that no competing interests exist.*  **Authors with competing interests**  Enter competing interest details beginning with this statement:  *I have read the journal's policy and the authors of this manuscript have the following competing interests: [insert competing interests here]* |  |
| * typeset |  |
| **Ethics Statement**  Enter an ethics statement for this submission. This statement is required if the study involved:   - Human participants - Human specimens or tissue - Vertebrate animals or cephalopods - Vertebrate embryos or tissues - Field research   Write "N/A" if the submission does not require an ethics statement.  General guidance is provided below. Consult the [submission guidelines](http://journals.plos.org/plosone/s/submission-guidelines#loc-guidelines-for-specific-study-types) for detailed instructions. **Make sure that all information entered here is included in the Methods section of the manuscript.** | N/A |

| **Format for specific study types**  **Human Subject Research (involving human participants and/or tissue)**   - Give the name of the institutional review board or ethics committee that approved the study - Include the approval number and/or a statement indicating approval of this research - Indicate the form of consent obtained (written/oral) or the reason that consent was not obtained (e.g. the data were analyzed anonymously)   **Animal Research (involving vertebrate animals, embryos or tissues)**   - Provide the name of the Institutional Animal Care and Use Committee (IACUC) or other relevant ethics board that reviewed the study protocol, and indicate whether they approved this research or granted a formal waiver of ethical approval - Include an approval number if one was obtained - If the study involved *non-human primates*, add *additional details* about animal welfare and steps taken to ameliorate suffering - If anesthesia, euthanasia, or any kind of animal sacrifice is part of the study, include briefly which substances and/or methods were applied   **Field Research**  Include the following details if this study involves the collection of plant, animal, or other materials from a natural setting:   - Field permit number - Name of the institution or relevant body that granted permission |  |
| --- | --- |
|  |  |
| **Data Availability**  Authors are required to make all data underlying the findings described fully available, without restriction, and from the time of publication. PLOS allows rare exceptions to address legal and ethical concerns. See the [PLOS Data Policy](http://journals.plos.org/plosone/s/data-availability) and [FAQ](http://journals.plos.org/plosone/s/data-availability#loc-faqs-for-data-policy) for detailed information. | Yes - all data are fully available without restriction |

| A Data Availability Statement describing where the data can be found is required at submission. Your answers to this question constitute the Data Availability Statement and **will be published in the article**, if accepted. |  |
| --- | --- |
| **Important:** Stating ‘data available on request from the author’ is not sufficient. If your data are only available upon request, select ‘No’ for the first question and explain your exceptional situation in the text box. |  |
| Do the authors confirm that all data underlying the findings described in their manuscript are fully available without restriction? |  |
| **Describe where the data may be found in full sentences. If you are copying our sample text, replace any instances of XXX with the appropriate details.**   - If the data are **held or will be held in a public repository**, include URLs, accession numbers or DOIs. If this information will only be available after acceptance, indicate this by ticking the box below. For example: *All XXX files are available from the XXX database (accession number(s) XXX, XXX.*). - If the data are all contained **within the manuscript and/or Supporting Information files**, enter the following: *All relevant data are within the manuscript and its Supporting Information files.* - If neither of these applies but you are able to provide **details of access elsewhere**, with or without limitations, please do so. For example:   *Data cannot be shared publicly because of [XXX]. Data are available from the XXX Institutional Data Access / Ethics Committee (contact via XXX) for researchers who meet the criteria for access to confidential data.*  *The data underlying the results presented in the study are available* | All relevant data are within the manuscript and its Supporting Information files. |

| *from (include the name of the third party and contact information or URL).*   - This text is appropriate if the data are owned by a third party and authors do not have permission to share the data.   * typeset |  |
| --- | --- |
| Additional data availability information: |  |

Manuscript	[Click here to access/download;Manuscript;Manuscript versie](https://www2.cloud.editorialmanager.com/pone/download.aspx?id=40132384&guid=a44e1893-104b-4513-98d1-0ef6e6aab65c&scheme=1)[<https://www2.cloud.editorialmanager.com/pone/download.aspx?id=40132384&guid=a44e1893-104b-4513-98d1-0ef6e6aab65c&scheme=1> 8.docx](https://www2.cloud.editorialmanager.com/pone/download.aspx?id=40132384&guid=a44e1893-104b-4513-98d1-0ef6e6aab65c&scheme=1)

## 1 Microbial drinking water quality deterioration during distribution and household

2	usage, determined together with citizen scientists.

3

4	Paul W.J.J. van der Wielen^1,^ ^2^* Stijn Brouwer^1^, Marco Dignum^3^, Merijn Schriks^1^

5

6 1 KWR Water Research Institute, Nieuwegein, The Netherlands
7 2 Department of Microbiology, Faculty of Science, Radboud Institute of Biological and
8 Environmental Science, Radboud University, Nijmegen, The Netherlands
9 3 Waternet, Amsterdam, The Netherlands.
10 *Corresponding author
11 E-mail: [paul.van.der.wielen@kwrwater.nl](mailto:paul.van.der.wielen@kwrwater.nl)

12

13	Short title: Drinking water quality during transport and storage

14	**Abstract**

15 During transport and storage of drinking water in the distribution system, premise plumbing
16 systems or containers used by consumers, the microbial water quality might deteriorate. Here,
17 ,we studied the vulnerability of non-chlorinated drinking water produced by two treatment plants
18 to deterioration, by involving citizen scientists. Citizen scientists in Amsterdam sampled their
19 drinking water directly from their kitchen tap after overnight stagnation, after flushing, and after
20 storage in containers like reusable plastic bottles. Subsequently, prokaryotic cell counts using
21 flow cytometry, ATP concentrations (which indicate the portion of the microbial biomass that is metabolically active) and the prokaryotic community composition were
22 determined in the laboratory. The results demonstrated that citizen scientists were able to
23 reliably sample drinking water. The microbiological parameters measured remained stable
24 during drinking water transport in the distribution system, whereas overnight stagnation in the
25 premise plumbing system could result in an increase or decrease of microbial biomass
26 parameters and slightly altered the prokaryotic composition. Drinking water storage in
27 containers dramatically increased microbial biomass, decreased bacterial diversity and shifted
28 the bacterial community composition. Furthermore, sampled drinking water from the
29 distribution system had a specific community composition related to either plant A or B, which
30 could be used to determine the origin of drinking water sampled from supposedly mixed zones
31 in the distribution system. Overall, we conclude that citizen scientists can reliably sample
32 drinking water for microbial analyses, that ATP and flow cytometry cell counts are poor
33 indicators for microbial regrowth in the distribution system and that drinking water is a product
34 prone to microbial deterioration during storage.

35

# 36 Introduction

37 In contrast to many other countries, drinking water in the Netherlands is distributed without a
38 disinfectant residual (1). Microbial growth in Dutch drinking water distribution and premise
39 plumbing systems are restricted by limiting the concentration of biodegradable organic carbon.
40 Despite the high quality drinking water in the Netherlands (2), microbial growth occurs during
41 transport of drinking water to the consumer in the distribution and premises plumbing
42 system (3-7), which can result in the growth of opportunistic pathogens that pose a threat to public
43 health, aesthetic complaints (occurrence of invertebrates visible to the human eye and
44 odour/taste) and technical complaints (pressure loss due to clogging of water meters with
45 invertebrate biomass or corrosion) (8). Some of these risks seem to be higher in the premises
46 plumbing system due to longer retention times and a higher surface-to-volume ratio and water
47 temperature (9). Due to the high quality of drinking water and absence of odour/taste issues
48 related to a disinfectant residual, it is common practice in the Netherlands that consumers refill
49 their containers (i.e. bottles, water cookers, coffee reservoirs, etcetera) with drinking water from
50 the tap. This offers both environmental and costs advantages as drinking water is much cheaper
51 than bottled water and the number of water bottles used in the Netherlands is very low compared
52 to other countries (10-12). However, the impact of storing drinking water in these bottles on the
53 microbial water quality remains unknown.
54 Different studies investigated the influence of drinking water distribution on active
55 microbial biomass, bacterial cell numbers and bacterial community in drinking water. Most of
56 these studies showed no influence of distribution on the concentration of active biomass (ATP),
57 bacterial cell numbers or the bacterial community composition in the drinking water,
58 irrespective of the source water used (groundwater versus surface water) or disinfection strategy
59 applied (chlorination, chloramination, or no disinfectant residual) (3, 6, 7, 13-18). In contrast,
60 others demonstrated an increase in cell numbers in drinking water during distribution or a
61 change in bacterial community composition (19, 20). Fewer studies have investigated the
62 influence of premises plumbing systems in private houses on active biomass, bacterial cell
63 counts and bacterial community composition. When drinking water with a disinfectant residual
64 was distributed, stagnation in premises plumbing system led to decreased concentration of the
65 disinfectant residual, a 2-log increase in cell numbers, a decreased bacterial diversity and
66 aberrant community composition (21, 22). In unchlorinated drinking water systems, it was
67 observed that active biomass (ATP) and bacterial cell numbers only slightly increased (< 1-log
68 increase) during overnight stagnation in the premises plumbing systems of different households
69 (23, 24). Due to the lack of a statistical data evaluation in those two studies, it cannot be
70 concluded whether overnight stagnation of unchlorinated drinking water in premises plumbing
71 system significantly affected the microbial biomass.
72 Drinking water quality in the Netherlands is routinely monitored by the drinking water
73 companies for legal microbial parameters related to regrowth (heterotrophic plate counts at
74 22°C (HPC), *Aeromonas* plate counts and *Legionella* plate counts) and recurrently for
75 additional non-legal parameters (e.g. ATP, AOC, biomass production potential, cell counts).
76 The microbial drinking water quality in the Netherlands has been extensively studied in the past
77 (8), and it was observed that general microbial drinking water quality parameters (e.g. ATP,
78 cell counts, *Aeromonas*, HPC), biological stability parameters (e.g. AOC, biofilm formation
79 rate) and bacterial community composition differs between different production locations (6, 7,
80 17, 25, 26). This implies that the effect of drinking water storage on the microbial water quality
81 might differ between drinking water treatment plants. None of the microbial data gathered from
82 these monitoring programs, however, is actively communicated to the drinking water
83 consumers. Not communicating such data to drinking water consumers might result in the
84 observed “gap” or “distance” between water (companies) and citizens (customers) that has been
85 reported to exist by a 2014 water governance assessment in the Netherlands (27). The new EU
86 Drinking Water Directive (2020/2184) emphasizes the importance of transparency regarding
87 water quality information. Aiming to increase and maintain consumer confidence in water
88 quality, it mandates that consumers must have access to up-to-date and comprehensible
89 information about their drinking water quality. Over the past decade, a “new dawn” of citizen
90 science can be witnessed, studies where the public actively participates in the generation of
91 science-based knowledge (28). One of the issues involving citizen scientists in microbial
92 drinking water quality is whether citizen scientists can provide reliable microbial sampling and
93 analysis of drinking water and, thus, if they can be involved in monitoring the drinking water
94 quality at the tap.
95 The goals of our research were to determine how vulnerable drinking water is to
96 deterioration and to evaluate the feasibility of involving citizen scientists in reliable monitoring
97 of the microbial drinking water quality. This was done by determining the influence of (i)
98 drinking water transport in the distribution system, (ii) overnight stagnation in the premise
99 plumbing system and (iii) storage in bottles on a range of microbial water quality parameters.
100 The research was conducted in collaboration with citizen scientists in the city of Amsterdam
101 and included households that retrieved drinking water from one of the two different treatment
102 plants that supply the city with drinking water, as well as households that retrieved a mixture
103 of drinking water from these two treatment plants. This approach made it possible to also
104 determine the effect of mixing drinking water types on the microbial water quality.

105

# 106 Materials and methods

107 **Sampling locations and citizen scientists**
108 Drinking water for Amsterdam is produced at two different locations. Treatment plant A treats
109 surface water using precipitation/coagulation, rapid sand filtration, dune infiltration, rapid sand
110 filtration, ozonation, softening, active carbon filtration and slow sand filtration and is
111 distributed in the Western part of Amsterdam. Treatment plant B treats surface water using
112 precipitation/coagulation, rapid sand filtration, ozonation, softening, active carbon filtration and
113 slow sand filtration and is distributed in the Eastern part of Amsterdam. The center part of the
114 city receives drinking water from plant A and B.
115 The recruitment and selection of citizen scientists has been described before (37) and
116 has resulted in the participation of 43 citizen scientists that lived across the inner city of
117 Amsterdam (Fig S1). Eighteen of them received drinking water from treatment plant A, 17 from
118 treatment plant B and 8 received drinking water from both treatment plants. The drinking water
119 samples were taken by the citizen scientists in June 2016. All of them sampled drinking water
120 at the kitchen tap in their household after overnight stagnation in the premises plumbing system
121 and were, thus, taken in the morning before drinking water was used for other purposes, e.g.
122 toilet flushing, showering, cleaning or drinking. They sampled drinking water directly after
123 opening the tap (first flush) and after flushing for 5 min, the latter represents a drinking water
124 sample from the distribution system. Next to these drinking water samples, the citizen scientists
125 were invited to take a water sample of their own interest. Thirty of the 43 citizen scientists
126 choose to sample drinking water that was stored in different containers (e.g. plastic bottles,
127 glass bottles, coffee machine reservoirs). Because the other 13 citizen scientists chose not to
128 sample drinking water, non-drinking water samples were omitted from the data analyses
129 presented in this publication. Furthermore, drinking water samples were also taken directly at
130 both treatment plants, but by trained personnel in June 2016. A small portion of the drinking
131 water samples were immediately used by the citizen scientists to determine bacterial and fungal
132 plate counts, whereas the remainder of the drinking water samples were stored and transported
133 to the lab at 4°C and were processed within 24 h. The ATP concentrations, cell numbers and
134 16S rRNA gene sequences were determined at the laboratory of KWR.

135

136 **Plate counts, ATP and cell numbers**
137 The citizen scientists determined plate counts by inoculating an agar dipslide for bacteria and
138 an agar dipslide for fungi (Sani-Check BF, Biosan Laboratories Inc, Warren, MI, USA) with
139 drinking water that was sampled after 5 min of flushing and with the stored drinking water
140 according to the manufacturer protocol. The dipslides were incubated for seven days at room
141 temperature, after which the colony-forming units were counted by the citizen scientists.
142 The total ATP concentration was determined in duplicate in all drinking water samples
143 by measuring the amount of light produced in the luciferine-luciferase assay as previously
144 described (6). In a previous study it was shown and explained that free ATP is negligible in
145 drinking water samples from the Netherlands (6), meaning that total ATP concentrations
146 determined in this study are a reliable measure for active biomass. The total and membrane-
147 intact cell counts were determined using a flow cytometer as described before (40).

148

149 **DNA isolation and community analysis**
150 The drinking water samples (500 ml) were vacuum filtered through polycarbonate track-edge
151 membrane filters with a diameter of 50 mm and a pore size of 0.2 µm (Sartorius; Goettingen,
152 Germany). Filters were transferred to a bead tube of the MoBio PowerBiofilm Kit (MoBio;
153 Carlsbad, USA) containing 350 µl of Solution BF1 of the kit. Samples were subsequently
154 processed to isolate DNA and remove PCR-inhibitors according to the suppliers protocol and
155 purified DNA was finally eluted in 100 µl elution buffer BF7.
156 Every drinking water sample was processed in duplicate to generate 16S rRNA gene
157 amplicons using previously described 515F and 806R primers (containing Illumina adapter
158 overhangs as described by Illumina) targeting the V4 hyper variable region of the 16S rRNA
159 gene (57). Triplicate amplicon reactions were pooled and 25 µl of this mixture was cleaned,
160 indexed and sequenced as described in the Illumina MiSeq 16S Metagenomic sequencing
161 library	preparation	protocol	(https://support.illumina.com/content/dam/illumina-
162 support/documents/documentation/chemistry_documentation/16s/16s-metagenomic-library-
163 prep-guide-15044223-b.pdf, June 2016). The final amplicon concentration loaded on the MiSeq
164 system was 4 pM supplemented with 10% PhiX (Illumina; San Diego USA) to add diversity.
165 Negative controls were included in every experiment to monitor the presence of contaminating
166 DNA. Version 1.37.0 of the MOTHUR software package (Schloss et al. 2009) was used to
167 process all MiSeq datasets using the procedure previously described (Kozich et al. 2013) and
168 summarized	in	the	MiSeq	standard	operating	procedure
169 [(http://www.mothur.org/wiki/MiSeq_SOP,](http://www.mothur.org/wiki/MiSeq_SOP) June 2016). The R1 sequences were quality filtered
170 by removing sequences containing ambiguous bases and sequences with an average quality
171 score below 35. Paired-end reads were first assembled into contigs. The quality of the quality
172 filtered R1 reads, and the assembled reads was further improved by (i) removing sequences
173 containing homopolymers of minimal 8 nt, (ii) removing sequences with ambiguous bases and
174 (iii) by using a 1% precluster error. The remaining sequences were aligned to the Silva 16S
175 RNA gene sequence database (Pruesse et al. 2007) and chimeric sequences were removed using
176 UCHIME (Edgar et al. 2011). Sequences were classified by comparison to the Ribosomal
177 Database Project sequences (Cole et al. 2009) using a minimal confidence score of 80%.
178 Sequences were first clustered into Operational Taxonomic Units (OTU) at a 3% dissimilarity
179 level definition and taxonomically classified using trainset 9 of the RDP Hierarchy Browser.
180 The online tool Microbiome Analyst, with default settings, was used for determining
181 bar graphs at the taxonomic level (Dhariwal et al., 2017). The latter was determined by
182 calculating the weighted UniFrac distance matrix, which was used to determine principal
183 coordinates analysis (PCoA) plots. The program PRIMER-e V7 [(www.primer-e.com)](http://www.primer-e.com/) was used
184 to determine differences in bacterial community composition between samples by calculating
185 Bray-Curtis dissimilarities, which were subsequently used as input for principle coordinates
186 analysis (PCoA). A permutational multivariate analysis of variance (PERMANOVA) was done
187 on the constrained axes used in ordination, using a p-value of 0.05 as cut-off value for statistical
188 significance.

189

190	**Results**

191 **Citizen Scientists**
192 In total 43 citizen scientists were selected in our study to participate in sampling and analyzing
193 drinking water from the kitchen tap of their household. The ATP concentrations of the drinking
194 water samples taken by the citizen scientists after 5 min flushing (representing drinking water
195 from the distribution system) and at the treatment plant are shown in Fig 1. The ATP
196 concentration in the treated water at plant A was 1.0 ng ATP/l and at plant B 4.3 ng ATP/l. The
197 ATP-concentrations in the flushed drinking water samples taken by the citizen scientists whose
198 houses were supplied with drinking water from the distribution system of plant A varied
199 between < 1.0 and 2.2 ng ATP/l with an average of 1.3 ± 0.8 ng ATP/l (Table 1). The ATP-
200 concentrations in the flushed drinking water samples taken by the citizen scientists whose
201 houses were supplied with drinking water from the distribution system of plant B varied
202 between < 1.0 till 3.5 ng ATP/l, with an average of 2.1 ± 1.0 ng ATP/l. These ATP
203 concentrations are well in the range of what is normally measured in drinking water from the
204 distribution system of both plants in Amsterdam (pers. comm. Marco Dignum). In addition,
205 none of the drinking water samples taken from the tap by the citizen scientists had aberrant cell
206 numbers, colony counts or bacterial community composition (data described below), compared
207 to the samples taken by other citizen scientists or to the samples taken at the treatment plant by
208 trained personnel.
209 ***Fig 1. The ATP concentrations of the drinking water sampled****. The drinking water samples*
210 *at treatment plant A and B (T) were sampled by professional laboratory personnel and the*
211 *drinking water samples at the tap were sampled by citizen scientists after 5 min flushing at the*
212 *kitchen tap in their household (supplied with drinking water from plant A, plant B or from*
213 *plant A and B, mix).*
214 **Table 1. Average and standard deviation of ATP concentration, intact cell numbers, total cell numbers, ATP per intact cell and colony**
215 **forming units of bacteria and fungi in drinking water from treatment plant A, treatment plant B and a mix of plant A and B.**

|  | | ATP (ng l^-1^) | | Intact cells (N ml^-1^) | | Total cells (N ml^-1^) | | ATP per cell (ng cell^-1^)^a^ | | Bacteria (cfu stick^-1^) | | Fungi (cfu stick2^-1^1)6 | |
| --- | --- | --- | --- | --- | --- | --- | --- | --- | --- | --- | --- | --- | --- |
| Type | Plant | Average | Stdev | Average | Stdev | Average | Stdev | Average | Stdev | Average | Stdev | Average | Stdev |
| Treated | A | 1.0 | - | 6.7×10^4^ | - | 7.3×10^4^ | - | 1.5×10^-8^ | - | - | - | - | 21- 7 |
| Flushed | A | 1.3 | 0.8 | 6.0×10^4^ | 1.1×10^4^ | 6.6×10^4^ | 1.4×10^4^ | 2.9×10^-8^ | 8.0×10^-9^ | 0.4 | 4.6 | 0.1 | 221.28 |
| Direct | A | 2.1 | 1.2 | 6.8×10^4^ | 1.4×10^4^ | 7.8×10^4^ | 1.6×10^4^ | 3.3×10^-8^ | 1.6×10^-8^ | - | - | - | -  219 |
| Stored | A | 96.2 | 139.0 | 2.7×10^5^ | 2.8×10^5^ | 4.7×10^5^ | 4.1×10^5^ | 4.8×10^-7^ | 9.7×10^-7^ | 259.0 | 21.9 | 5.8 | 308.3 |
| Treated | B | 4.3 | - | 9.2×10^4^ | - | 1.1×10^5^ | - | 4.6×10^-8^ | - | - | - | - | 22- 0 |
| Flushed | B | 2.1 | 1.0 | 8.4×10^4^ | 1.9×10^4^ | 1.1×10^5^ | 7.0×10^4^ | 2.8×10^-8^ | 8.6×10^-9^ | 0.4 | 6.7 | 0.2 | _2_3_2_.6_1_ |
| Direct | B | 2.9 | 1.8 | 1.1×10^5^ | 6.6×10^4^ | 1.7×10^5^ | 1.6×10^5^ | 2.9×10^-8^ | 1.3×10^-8^ | - | - | - | - |
| Stored | B | 64.5 | 76.1 | 3.0×10^5^ | 3.5×10^5^ | 4.1×10^5^ | 5.1×10^5^ | 2.8×10^-7^ | 2.1×10^-7^ | 64.2 | 36.2 | 0.3 | 2_5_2.42 |
| Flushed | M | 1.1 | 0.7 | 5.6×10^4^ | 1.0×10^4^ | 6.2×10^4^ | 1.2×10^4^ | 2.7×10^-8^ | 8.2×10^-9^ | 0.9 | 5.2 | 0.2 | 232.63 |
| Direct | M | 1.7 | 0.9 | 6.0×10^4^ | 1.7×10^4^ | 6.9×10^4^ | 2.1×10^4^ | 3.4×10^-8^ | 1.9×10^-8^ | - | - | - | -  224 |
| Stored | M | 20.1 | 31.6 | 9.5×10^4^ | 5.9×10^4^ | 1.2×10^5^ | 7.0×10^4^ | 1.9×10^-7^ | 1.9×10^-7^ | 4.0 | 8.9 | 0.2 | 5.2 |
|  |  |  |  |  |  |  |  |  |  |  |  |  | 225 |

226 **Influence of distribution, overnight stagnation and storage of drinking water**
227 The ATP concentration remained relatively stable during distribution of the drinking water from
228 treatment plant A to the households in the distribution system of this plant (Table 1, Fig 2). In
229 addition, the average intact and total cell numbers were slightly lower in the distributed
230 compared to the treated water of plant A. The ATP concentration in the treated water of plant
231 B was twice as high as the average ATP concentration in the distributed drinking water from
232 this plant and at all locations the distributed drinking water contained lower ATP concentrations
233 than in the treated water (Table 1, Fig 2). Furthermore, the intact and total cell numbers were
234 comparable between treated and distributed drinking water from plant B (Table 1).
235 The average ATP concentration, membrane-intact and total cell numbers were higher in
236 drinking water sampled directly from the tap after overnight stagnation than in drinking water
237 sampled at the tap after 5 min flushing (Table 1). However, the influence of overnight
238 stagnation on ATP or bacterial cell numbers differed considerably per location. At 25 of the 43
239 locations the ATP concentration was higher in drinking water directly sampled from the tap
240 after overnight stagnation in the premises plumbing system than in drinking water sampled
241 from the tap after 5 min flushing (Fig 2). At 19 of these 25 locations the ATP increased with
242 more than 20%, indicating microbial growth in the premises plumbing system during overnight
243 stagnation at these 19 locations. Furthermore, at 15 locations the ATP concentrations were
244 lower in the directly tapped drinking water samples than in the flushed drinking water samples
245 with eight locations showing a more than 20% decrease in ATP concentration. The remaining
246 three locations showed the same ATP concentration in directly tapped and flushed drinking
247 water.

### 248 Fig 2. ATP-concentration in drinking water sampled at two plants and 43 locations in the

249 ***distribution systems.*** *Drinking water came from Plant A (blue symbols), Plant B (yellow*
250 *symbols), mixed zone of Plant A and B (red symbols) or was treated water sampled at plant A*
251 *or B (green symbols). Symbols: ◆, Drinking water sampled at the kitchen tap after 5 min*
252 *flushing (representing drinking water from the distribution system); ■, drinking water*
253 *sampled directly after opening the kitchen tap after overnight stagnation; ▲, drinking water*
254 *stored in bottles. Open symbols: ATP concentrations lower than detection limit of 1 ng/l ATP.*
255 The number of total and membrane-intact cells and total cells was higher in directly
256 tapped drinking water samples after overnight stagnation than in the 5 min flushed drinking
257 water samples in 37 (total cells) or 35 (membrane-intact cells) of the 43 samples, but only at
258 around ten locations the membrane intact or total cell numbers increased with more than 20%
259 (Fig S2 and S3). At four (total cells) to six (membrane-intact cells) locations, intact and total
260 cell numbers were lower in directly tapped drinking water samples than in flushed samples, but
261 only at one location was this difference more than 20%. The remaining two locations showed
262 the same membrane-intact and total cell numbers in directly tapped and flushed drinking water
263 samples.
264 The average ATP concentration and cell numbers were substantially higher in the
265 drinking water samples that have been stored in containers than in drinking water sampled after
266 5 min flushing at the tap (Table 1, Fig 2, S2 and S3). On average the ATP increased by a factor
267 12 to 74, whereas the total or intact cell numbers only increased by 1.6 to 7.2. As a result, the
268 ATP per cell ratio was much higher in stored drinking water than in flushed drinking water.
269 Practically all locations showed more than 20% higher ATP concentrations or cell numbers in
270 stored drinking water than in flushed drinking water samples from the tap, demonstrating
271 microbial growth occurs during drinking water storage in different containers. The results from
272 the bacterial and fungal plate counts that have been determined by the citizen scientists,
273 demonstrated that stored drinking water also had higher average bacterial and fungal plate
274 counts than drinking water sampled after 5 min flushing of the tap (Table 1). Particularly the
275 increase in bacterial plate counts in stored drinking water compared to flushed drinking water
276 from the tap was much larger (up to 724 times) than observed for ATP and especially cell
277 numbers counted with flow cytometer. This result is an indication that not only the biomass
278 increased during drinking water storage but the community also shifted towards a
279 one harboring more bacteria capable of growing on an agar medium.
280 The bacterial community composition was elucidated using microbial profiling of the
281 bacterial 16S rRNA gene sequences obtained with next generation sequencing. The number of
282 sequences analyzed, OTUs observed, and the Shannon diversity indices are shown in Table S1
283 (for each sample) and Table 2 (average values per water type and plant). In general, 25,000 to
284 95,000 16S rRNA gene sequences were analyzed per sample and the average sequences
285 obtained did not differ noticeably per water type and treatment plant. The observed OTU
286 numbers and the Shannon diversity index were highest in drinking water sampled after 5 min
287 flushing of the tap and were slightly lower in drinking water samples that were taken directly
288 from the tap after overnight stagnation (Table 2). A dramatic decrease in OTU numbers and
289 Shannon index was observed in the drinking water samples that were stored in containers (Table
290 2).

291

292 **Table 2. Average and standard deviation of number of sequences and OTUs, and**
293 **Shannon index for the different drinking water types from treatment plant A, treatment**
294 **plant B and a mix of plant A and B.**

\# Sequences	# OTUs	Shannon in2d9e5x Type	Plant	Average	Stdev	Average	Stdev	Average	Stdev

Treated	A	64058	3640	6.9

296

Flushed	A	54406	10239	3641	421	7.0	0.1

Direct	A	55353	13706	3055	474	6.5

2_0_9.7_3_

Stored	A	64390	19207	822	451	3.1	0.8

Treated	B	25060	2394	6.8

Flushed	B	53547	20532	3567	869	6.9

298

_2_0_9_._9_3

Direct	B	58871	16111	3032	934	6.2	1.2

Stored	B	67215	21229	2285	1383	4.8	_3_1_0_._0_8

Flushed	M	50905	18683	3440	675	7.1	0.1

Direct	M	50863	11898	2862	262	6.5

300.13

Stored	M	58878	30760	1247	1042	4.1	1.2

302

303

304 The relative abundance of the different dominant bacterial classes did not change
305 considerably during transport in the distribution system from the treatment plant to the
306 households of the citizen scientists (Fig 3A). Bacteria belonging to unclassified taxonomic
307 classes prevailed in these samples, indicating that a large part of the bacterial community
308 remained unknown even at the class level. Besides these unclassified classes, *Proteobacteria*
309 and *Acidobacteria* GP6 also dominated the treated and distributed drinking water samples.
310 Overnight stagnation in the premise plumbing system did not have a large impact on the
311 bacterial community at class level either (Fig 3B), although the relative abundance of bacteria
312 belonging to unclassified classes and *Acidobacteria* GP6 slightly decreased compared to the
313 flushed drinking water samples. This decrease was accompanied with an increase in relative
314 abundance of *Proteobacteria* classes and *Actinobacteria*. A dramatic change in the bacterial
315 community at class level was observed in the drinking water samples that were stored in
316 containers (Fig 3C). Compared to the flushed or directly sampled drinking water from the tap,
317 the relative abundance of bacteria belonging to unclassified classes in these stored drinking
318 water samples were very low, whereas *Proteobacteria* strongly dominated most of the stored
319 drinking water samples. Furthermore, the relative abundance of *Sphingobacteria* and
320 Actinobacteria also increased in several stored drinking water samples.

### 321 Fig 3. Taxaplot of the prokaryotic community members at class level observed in the

322 ***drinking water samples.*** *The drinking water samples were taken from the tap after 5 min*
323 *flushing (A), directly after opening the tap after overnight stagnation (B) and after drinking*
324 *water was stored in a container (C). Drinking water samples were retrieved from the*
325 *distribution area of Plant A, Plant B and the mixed zone of Plant A and plant B.*

326

327 The bacterial drinking water communities were also compared at OTU level using
328 principal coordinate analysis of the calculated Bray Curtis weight distance matrix. A
329 comparison of all analyzed drinking water samples (treated, flushed, directly tapped and stored
330 drinking water) showed that the bacterial community in treated, flushed and directly tapped
331 drinking water clustered more together compared to the stored drinking water samples (Fig 4A).
332 PERMANOVA showed that the bacterial community in the stored drinking water samples were
333 significantly different from those in the drinking water sampled at the plant or the tap. In
334 addition, the stored drinking water samples scattered over the whole PCoA plot, irrespective of
335 the source of the drinking water (treatment plant A, B or a mix of plant A and B). This
336 demonstrates that extensive changes occurred in the bacterial OTU community composition
337 when drinking water is stored in containers, but also that these changes are rather specific for
338 each household that stored the drinking water.
339 To better observe changes in the bacterial OTU community during drinking water
340 transport in the distribution system and overnight stagnation in the premises plumbing system,
341 a PCoA plot was also made without the stored drinking water samples (Fig 4B). These results
342 showed that the bacterial community also differed between drinking water taken directly from
343 the tap or after 5 minutes flushing and PERMANOVA confirmed that these changes were
344 statistically significant (p<0.05). Furthermore, we observed that the bacterial community
345 composition of the treated water at plant A is comparable to the bacterial community
346 composition in all drinking water samples from the distribution system (i.e. flushed samples)
347 where drinking water from plant A is distributed. Similar results were observed for the flushed
348 drinking water samples from the distribution system and the treated water of plant B. These
349 results, thus, demonstrate that the bacterial OTU community composition remained stable
350 during transport in the distribution system of plant A and B. Furthermore, the drinking water
351 samples originating from plant A clustered in general separate from the drinking water samples
352 originating from plant B. In contrast, the bacterial community in drinking water sampled from
353 households where drinking water either came from plant A or plant B clustered with the
354 drinking water samples originating from plan A. The pairwise PERMANOVA showed that the
355 community composition of drinking water originating from plant B were significantly different
356 from those in drinking water originating from plant A or in drinking water sampled from
357 households that either received drinking water from plant A or plant B (p<0.05).

### 358 Fig 4. Principal Coordinates Analysis (PCoA) plot of the OTU composition in the drinking

359 ***water samples.*** *Drinking water samples from the treatment plant (green) and drinking water*
360 *taken directly (red) or after 5 min flushing (blue) at the tap and after storage in containers*
361 *(yellow). The drinking water came from treatment plant A, treatment plant B or were taken at*
362 *a mixed zone in the distribution system where drinking water came from plant A and B. Panel*
363 *A: all samples; Panel B: stored drinking water samples are omitted.*

364

365 The PCoA results also showed that the flushed and directly tapped drinking water
366 samples clustered based on the origin of the water (treatment plant A or B) at the first coordinate
367 and on water type (flushed or directly tapped) at the second coordinate (Fig 4B). This indicates
368 that the origin of the drinking water was a stronger driver for changes in the bacterial OTU
369 community composition than overnight stagnation in the premises plumbing system. Still, the
370 results clearly showed that changes in the bacterial OTU community composition were larger
371 during overnight stagnation in the premises plumbing system of a household, than during
372 transport in the distribution system from the treatment plant to the water meter (i.e. inlet) of the
373 household.
374 The overall results from the community analysis, thus, demonstrated a dramatic change
375 in the community composition, OTU numbers and the Shannon diversity during storage and
376 use of drinking water in containers, which was also accompanied in a change from unclassified
377 to classified bacterial classes. We, therefore, also examined if drinking water storage resulted
378 in an increase in highly dominant (≥ 10% relative abundance) OTUs in the bacterial community
379 and, if so, to what genera these OTUs belonged (Table S2). An OTU with a relative abundance
380 above 10% in the drinking water sample taken after 5 min flushing or directly at the tap was
381 only observed at respectively three or ten of the 30 households where drinking water was
382 sampled after flushing, overnight stagnation and storage. In contrast, 58 OTU(s) with a relative
383 abundance above 10% were observed in stored drinking water at 26 of the 30 households, with
384 most of these samples (19 out of these 26 households) showing more than one OTU having a
385 relative abundance above 10%. In some instances, the same OTU dominated in containers from
386 different locations, resulting in a total of 36 unique OTUs that had a relative abundance of more
387 than 10%.
388 Twenty-one of the 58 OTUs observed in stored drinking water with a relative abundance
389 above 10% were not observed in the flushed or directly tapped drinking water at the same
390 location (Table S2). This indicates that these bacteria were absent or only present at very low
391 numbers in the drinking water used to fill the containers before storage, and that they grew to
392 dominant levels during. In addition, 20 other of these 58 OTUs in stored drinking water already
393 slightly increased in relative abundance during overnight stagnation in the premises plumbing
394 system, but in general this increase was negligible compared to the large increase during
395 drinking water storage. Still, it might demonstrate that growth of these bacteria already occurred
396 during overnight stagnation and continued when the drinking water was stored for a longer
397 period in a container. Three OTUs that had a relative abundance above 10% in stored drinking
398 water were observed in four different stored water samples, two OTUs in three different stored
399 waters samples and nine in two different stored water samples. The rest (22 OTUs) were only
400 observed in one of the stored drinking water samples. This result demonstrates that in each
401 stored drinking water in general (a) different OTU(s) dominated.
402 Most of the dominant OTUs observed in stored drinking water could be assigned to the
403 genus level (21 out of 36)(Table S2). The remaining OTUs could be assigned to the family (8
404 OTUs), order (4 OTUs) or class (3 OTUs) level. The dominant OTUs that could be assigned to
405 the genus level belonged to 15 different genera with *Acinetobacter* (5 OTUs) and *Pseudomonas*
406 (3 OTUs) being most often observed. At six households, drinking water storage in a container
407 resulted in a single OTU having a relative abundance above 50%. At two of these six households
408 the same OTU prevailed in stored drinking water and this OTU belonged to the genus
409 *Enhydrobacter*. In both households the relative abundance of this OTU already increased to
410 13.4% or 1.5% in drinking water during overnight stagnation, indicating that growth of this
411 OTU started during drinking water stagnation. At the other four households the prevailing OTU
412 in stored drinking water belonged either to the genus *Acidovorax*, *Acinetobacter*,
413 *Methylobacterium* or *Pseudomonas*. These OTUs had not yet increased significantly in the
414 drinking water during overnight stagnation and, thus, started to become dominant during
415 storage of the drinking water.

416

417 **Influence of treatment plant**
418 The ATP concentration and intact and total cell numbers in the treated water of plant A was
419 lower than that of plant B (Table 1). Moreover, the average ATP concentration and intact and
420 total cell numbers in drinking water taken after 5 min of flushing were significantly higher in
421 drinking water samples taken at households in the distribution system of plant B than taken at
422 households in the distribution system of plant A (p<0.05; one-way Anova with Bonferroni
423 posthoc). These results showed that, although differences in ATP and cell numbers were low
424 between drinking water samples from treatment plant A and plant B, the microbial water quality
425 at treatment plant A was slightly better than at treatment plant B. Furthermore, the drinking
426 water samples taken in the mixing zone of the distribution system, where drinking water from
427 plant A and B were distributed, had ATP concentrations and cell numbers that were
428 significantly different from samples taken in the distribution system of plant B (p<0.05), but
429 not from plant A (p>0.05). This result implies that the mixing zone of plant A and B were
430 mostly fed with drinking water from treatment plant A. In addition, it was observed that the
431 colony forming units of bacteria and fungi in flushed drinking water were in general below the
432 detection limit (1 cfu per dipslide) and, as a result, differences between the water types (i.e.
433 drinking water from plant A, plant B or mixing zone) could not reliably be determined for these
434 plate counts.
435 The relative abundance of 16S rRNA gene sequences belonging to the 14 most dominant
436 classes showed that the bacterial community did not differ considerably at class level between
437 drinking water sampled from the distribution system of plant A and B after 5 min flushing of
438 the tap (Fig 3A). Only the relative abundance of Acidobacteria GP6 was higher in flushed
439 drinking water from plant A compared to plant B, whereas the abundance of the β-Proteobaceria
440 was lower. Most of the flushed drinking water samples from the mixing zone of the distribution
441 system also seemed to have a lower relative abundance of β-Proteobacteria. The PCoA and
442 PERMANOVA of the distance matrix (based on Bray Curtis) at OTU level showed that the
443 bacterial OTU community composition of drinking water sampled from the distribution area of
444 plant A was significantly different from plant B (p<0.05) (Fig 4B). However, the bacterial OTU
445 community composition of distributed drinking water sampled from the mixing zone of the
446 distribution system was remarkably similar to the bacterial OTU community composition of
447 plant A and no statistically significant differences between the bacterial communities could be
448 observed (p>0.05). This result indicates again that drinking water from treatment plant A, but
449 not from treatment plant B, was distributed in the mixed zone of the distribution system when
450 the distributed drinking water samples were taken in the morning at the tap.

451

452	**Discussion**

453 **Citizen scientists**
454 Our study involved citizen scientists for sampling the different drinking water types and
455 determining the plate counts of bacteria and fungi from the sampled drinking water. The
456 collaboration with citizen scientists in water quality research is not new, with numerous
457 examples of citizen involvement, particularly in determining surface water quality (e.g. 29,
458 30). However, publications on the involvement of citizen scientists in monitoring drinking
459 water quality are scarce (31-36). In one of those studies, basic chemical parameters as pH,
460 conductivity, chloride, nitrate and hardness were measured by trained students that sampled the
461 drinking water in their homes (36). The student results were within the limits of uncertainties,
462 but for some parameters significant differences were observed with the laboratory results. In a
463 study where citizen scientists monitored the microbial water quality, they were recruited to
464 sample biofilms from the showerhead in their homes and to analyze basic chemical parameters
465 (31). These authors did not elaborate on whether and how involved citizen scientists were
466 trained and on the sampling and chemical data reliability of the citizen scientists. Low standard
467 deviations for the chemical parameter means were observed, but high variability between
468 samples for the microbial community analysis occurred. As a result, it remains difficult to
469 conclude whether involvement of citizen scientists resulted in the collection of reliable
470 microbial data in that study.
471 In our study, we instructed the citizen scientists by an instruction video that was shown
472 during an initial meeting, which was also accessible via a webpage dedicated to this project.
473 We compared the results from samples taken at each household by citizen scientists with the
474 results from samples taken at the treatment plant by a professional sampling technician, as well
475 as between citizen scientists. These comparisons clearly showed that no aberrant results for
476 ATP, cell numbers, heterotrophic plate counts, and community composition were obtained for
477 the drinking water samples taken after five minutes flushing by each of the 43 citizen scientists.
478 Furthermore, the ATP concentrations and cell numbers are in the range that the drinking water
479 company measures in these distribution systems with their water quality monitoring program.
480 We, therefore, conclude that, when properly instructed, citizen scientists can reliably sample
481 and analyze drinking water. In addition, a majority of the citizen scientists involved in our study
482 reported an increase in their confidence in both the drinking water quality and the drinking
483 water company as a result of their participation in this project (37). Based on these findings, we
484 encourage drinking water companies to consider collaborating with voluntary citizen scientists
485 for drinking water sampling and monitoring, since this opens possibilities to improve
486 monitoring strategies for drinking water quality while simultaneously increasing consumer
487 confidence in the safety and reliability of their drinking water.

488

489 **Drinking water residence in distribution and premise plumbing systems**
490 We investigated how the microbial quality of drinking water changed during distribution,
491 overnight stagnation in a premise plumbing system and storage in a container. The results
492 demonstrated that the active biomass, cell numbers and community composition remained
493 relatively stable during transport in the drinking water distribution system. Others have also
494 reported that the active biomass concentration, bacterial cell numbers or the bacterial
495 community composition in drinking water were hardly affected during transport in the
496 distribution system, irrespective of source water (groundwater versus surface water) and
497 disinfection strategy (chlorination, chloramination or no disinfectant residual) used (3, 6, 7, 13-
498 18). One might infer from these findings that regrowth in drinking water during distribution is
499 a negligible process.	However, this would be a misconception, as several studies have
500 demonstrated biofilm formation and growth of specific microorganisms in drinking water
501 during transport in the distribution system (4, 7, 38-43). Studies have also shown that some
502 opportunistic pathogens and heterotrophic and *Aeromonas* plate counts increased in drinking
503 water during transport in the distribution system of Amsterdam (40, 44). Consequently, there
504 seems to be an apparent difference between the dynamics in the generic microbiological
505 parameters and the dynamics of specific nuisance microorganisms during distribution of
506 drinking water in Amsterdam. This discrepancy between dynamics in general and specific
507 microbiological parameters have also been reported for drinking water in the distribution
508 systems of 28 different treatment plants in the Netherlands (7). As a result, the reliability of
509 monitoring generic microbiological parameters, like ATP, total cell count, membrane intact cell
510 counts and HNA/LNA cell counts as indicators for regrowth of nuisance organisms in drinking
511 water distribution systems can be questioned. Consequently, shifting routine microbial drinking
512 water quality monitoring from HPC, *Aeromonas* and *Legionella* plate counts to ATP or cell
513 counts using flow cytometry, as suggested previously (45, 46), is, therefore, not recommended.
514 It still also presses the need to develop other rapid indicators for regrowth problems in drinking
515 water distribution systems, since plate counts methods are time consuming.
516 Storage of drinking water had the largest influence on the microbial quality, as
517 evidenced by the dramatic changes in biomass, cell numbers and community composition when
518 drinking water was stored in containers. An onset of these significant changes could already be
519 observed during overnight stagnation, which led to a modest increase in biomass and cell
520 numbers and slightly affected the bacterial community composition at several households
521 analyzed in our study. Many studies have shown that stagnation of drinking water resulted in
522 altered microbial community and increased the risk of growth of opportunistic pathogens like
523 *L. pneumophila* or certain nontuberculous mycobacterial species (e.g. (9, 23, 47-52)). Most of
524 these studies, however, were conducted on drinking water with a disinfectant residual. In those
525 cases, stagnation leads to loss of disinfectant residual and, as a result, microbial growth is no
526 longer inhibited. Since drinking water in the Netherlands is distributed without a disinfectant
527 residual, the results from those studies cannot be compared with our study. Some studies have
528 also investigated the effect of (overnight) stagnation on the microbial quality of drinking water
529 without a disinfectant residual (23, 51). Those studies showed that the biomass and cell numbers
530 in drinking water increased, and that the microbial community composition slightly changed
531 during residence in the premise plumbing system. The latter observation was also made in our
532 study, but in contrast to those two studies we observed that it depended on the household
533 whether the biomass and cell numbers increased or decreased in the drinking water during
534 overnight stagnation in the premise plumbing system. This apparent discrepancy might have
535 been caused by the low number of households investigated in the previous studies (n=1 or n=10)
536 and households where drinking water biomass and cell numbers decreased during overnight
537 stagnation might have been missed.
538 The decrease in biomass and cell numbers we observed at several households might
539 have been caused by the low BDOC concentrations in the drinking water in the Netherlands
540 (25), resulting in microbial starvation during overnight stagnation. However, if this mechanism
541 would have been the cause behind the biomass decrease during overnight stagnation, biomass
542 should have decreased at all households, since they all receive drinking water with low BDOC
543 concentrations. Another possibility is that the households where a decrease in biomass and cell
544 numbers was observed after overnight stagnation, have a copper-based premise plumbing
545 system. It is known that copper ions can act as a disinfectant (53) and hence copper ions released
546 in the biofilm and water might have elucidated microorganisms in the biofilm and drinking
547 water. It is still important to stress that at several households biomass and cell numbers
548 increased in the drinking water during overnight stagnation, which demonstrates that overnight
549 stagnation can result in deterioration of the drinking water quality.

550

551 **Microbial drinking water quality deterioration during container storage**
552 Due to the good drinking water quality and the distribution without a disinfectant residual, it is
553 common practice in the Netherlands to consume water directly from the tap instead of bottled
554 water. Many people in the Netherlands do this by refilling a (plastic) container with drinking
555 water and, therefore, it was not surprising that for their own chosen water sample, most citizen
556 scientists sampled a container they use to refill with drinking water. The results for the drinking
557 water from these containers strikingly showed that there was a strong increase in biomass and
558 cell numbers concomitant with a decrease in diversity of the microbial community composition.
559 It can be concluded that container storage had the largest influence on the microbial water
560 quality compared to drinking water transport in the distribution system or overnight stagnation
561 in the premise plumbing system. A likely cause for the large impact on the microbial drinking
562 water quality during container storage is that people directly drink from these containers,
563 resulting in transfer of saliva from the mouth into the container. Such saliva transfer results in
564 introduction of bacteria from the oral microbiome and of relatively high BDOC concentrations
565 in the water, thereby promoting growth of bacterial species with a high maximum specific
566 growth rate. Our results showed that species belonging to the genus *Acidovorax*, *Acinetobacter*,
567 *Pseudomonas*, *Methylobacterium* and *Enhydrobacter* (Table S2) could strongly dominate (>
568 50% of the community) the drinking water in these containers. It has been demonstrated that
569 *Pseudomonas* species in drinking water have a relatively high maximum specific growth rate
570 but low affinity compared to other drinking water microorganisms (54), which further support
571 this hypothesis. Although storage of drinking water in these containers altered the microbial
572 water quality, it remains questionable whether these alterations would also have a health impact.
573 The results demonstrated that most of the genera that became dominant in these containers do
574 not contain virulent pathogenic species, suggesting that the public health risk of the drinking
575 water deterioration during storage in these containers was low.
576 To our knowledge, no previous studies have been published on the influence of drinking
577 water storage in (bottle) containers on the microbial community. However, several studies have
578 investigated dynamics in the microbial community of bottled mineral water during storage (48-
579 52). The results from these studies show that storage of bottled water generally also results in
580 increasing cell numbers and colony counts as well as a dramatic change in bacterial community
581 composition. In addition, Sala-Comorera et al. (51) observed that during storage of different
582 brands of bottled waters the percentage of cultivable bacteria increased with a sharp decrease
583 in diversity, which is in concordance with our observation for unchlorinated drinking water
584 stored in a container. It is not surprising that results from bottled mineral water storage were
585 comparable to our findings, since mineral water and drinking water in the Netherlands have low
586 BDOC concentrations and do not contain a disinfectant residual. In contrast to our study,
587 however, Sala-Comorera et al. (51) observed that most 16S rRNA gene sequences could be
588 identified to the genus level directly after bottling, but that during storage unclassified genera
589 became dominant.
590 Overall, we conclude from our results that citizen scientists can reliably sample drinking
591 water for microbial analyses, and we recommend their involvement when sampling drinking
592 water from households. Furthermore, generic microbial parameters like ATP concentration or
593 cell counts determined by flow cytometry seem to be less suitable indicators for regrowth in the
594 distribution system, whereas the microbial community composition can reliably be used to track
595 the drinking water origin to a treatment plant. Finally, we conclude that drinking water is a
596 product prone to microbial quality deterioration during storage, comparable to other food
597 products (e.g. vegetables, dairy products, meat) (55, 56). Quality deterioration during other food
598 product storage can result in human health risks (due to growth of pathogens) or taste and odor
599 issues (55, 56). Whether microbial quality deterioration during drinking water storage in
600 containers can result in human health risks or taste and odor issues has not yet been investigated
601 to the best of our knowledge, nor can they be inferred from our study. A more targeted approach,
602 using specific detection methods for nuisance organisms, is needed before more definite
603 conclusions can be drawn about possible public health or aesthetic impacts of drinking water
604 storage in containers.

605

# 606 Acknowledgements

607 The authors would like to thank the citizen scientists that participated in this project, Maarten
608 Claassen and Leon Kors from Waternet for their help and discussions, and the staff of the
609 microbiological laboratory of KWR for analyzing the samples. A major part of the outcomes
610 presented in this paper was based on research financed by the Joint Research Program of the
611 drinking water companies in The Netherlands.

612

613	**References**

614	1.	Smeets PWMH, Medema GJ, van Dijk JC. The Dutch secret: how to provide safe

615	drinking water without chlorine in the Netherlands. Drink Water Eng Sci. 2009; 2: 1-

616	14.

617	2.	Rosario-Ortiz F, Rose J, Speight V, von Gunten U, Schnoor J. How do you like your

618	tap water? Science 2016; 351: 912-914.

619 3.	Liu G, Bakker GL, Li S, Vreeburg JH, Verberk JQ, Medema GJ, Liu WT, Van Dijk
620 JC. Pyrosequencing reveals bacterial communities in unchlorinated drinking water
621 distribution system: an integral study of bulk water, suspended solids, loose deposits,
622 and pipe wall biofilm. Environ Sci Technol. 2014; 48: 5467-5476.
623 4.	Liu G, Tao Y, Zhang Y, Lut M, Knibbe W-J, van der Wielen P, Liu W, Medema G,
624 van der Meer W. Hotspots for selected metal elements and microbes accumulation and
625 the corresponding water quality deterioration potential in an unchlorinated drinking
626 water distribution system. Water Res. 2017; 124: 435-445.
627 5.	Prest EI, Schaap PG, Besmer MD, Hammes F. Dynamic hydraulics in a drinking
628 water distribution system influence suspended particles and turbidity, but not
629 microbiology. Water. 2021; 13. DOI: 10.3390/w13010109.
630 6.	van der Wielen PWJJ, van der Kooij D. Effect of water composition, distance and
631 season on the adenosine triphosphate concentration in unchlorinated drinking water in
632 the Netherlands. Water Res. 2010; 44: 4860-4867.
633 7.	van der Wielen PWJJ, Bakker G, Atsma A, Lut M, Roeselers G, de Graaf B. A survey
634 of indicator parameters to monitor regrowth in unchlorinated drinking water. Environ
635 Science: Water Res Technol. 2016; 2: 683-692.
636 8.	van der Kooij D, van der Wielen PWJJ. Microbial growth in drinking-water supplies.
637 Problems, causes, control and research needs. IWA Publishing, London, UK. 2014.
638 9.	Flemming HC, Bendinger B, Exner M, Gebel J, Kistemann T, Schaule G, Szewzyk U,
639 Wingender J. The last meters before the tap: where drinking water quality is at risk. In:
640 van der Kooij D, van der Wielen PWJJ, editors, Microbial Growth in Drinking-Water
641 Supplies Problems, Causes, Control and Research Needs. IWA Publishing, London,
642 UK; 2014. pp. 207-238.
643 10.	Botto S, Niccolucci V, Rugani B, Nicolardi V, Bastianoni S, Gaggi C. Towards lower
644 carbon footprint patterns of consumption: The case of drinking water in Italy. Environ
645 Science Pol. 2011; 14: 388-395.
646 11.	Fantin V, Scalbi S, Ottaviano G, Masoni P. A method for improving reliability and
647 relevance of LCA reviews: The case of life-cycle greenhouse gas emissions of tap and
648 bottled water. Sci Total Environ. 2014; 476-477: 228-241.
649 12.	Tosun J, Scherer U, Schaub S, Horn H. Making Europe go from bottles to the tap:
650 Political and societal attempts to induce behavioral change. WIREs Water. 2020; 7:
651 e1435.
652 13.	Eichler S, Christen R, Holtje C, Westphal P, Botel J, Brettar I, Mehling A, Hofle MG.
653 Composition and dynamics of bacterial communities of a drinking water supply
654 system as assessed by RNA- and DNA-based 16S rRNA gene fingerprinting. Appl
655 Environ Microbiol. 2006; 72:1858-1872.
656 14.	Henne K, Kahlisch L, Brettar I, Höfle MG. Analysis of structure and composition of
657 bacterial core communities in mature drinking water biofilms and bulk water of a
658 citywide network in Germany. Appl Environ Microbiol. 2012; 78: 3530-3538.
659 15.	Hwang C, Ling F, Andersen GL, LeChevallier MW, Liu W-T. Microbial community
660 dynamics of an urban drinking water distribution system subjected to phases of
661 chloramination and chlorination treatments. Appl Environ Microbiol. 2012; 78: 7856-
662 7865.
663 16.	Pinto AJ, Xi C, Raskin L. Bacterial community structure in the drinking water
664 microbiome is governed by filtration processes. Environ Sci Technol. 2012; 46: 8851-
665 8859.
666 17.	Roeselers G, Coolen J, van der Wielen PW, Jaspers MC, Atsma A, de Graaf B,
667 Schuren F. Microbial biogeography of drinking water: patterns in phylogenetic
668 diversity across space and time. Environ Microbiol. 2015; 17: 2505-2514.
669 18.	Vital M, Dignum M, Magic-Knezev A, Ross P, Rietveld L, Hammes F. Flow
670 cytometry and adenosine tri-phosphate analysis: Alternative possibilities to evaluate
671 major bacteriological changes in drinking water treatment and distribution systems.
672 Water Res 2012; 46: 4665-4676.
673 19.	Nescerecka A, Juhna T, Hammes F. Identifying the underlying causes of biological
674 instability in a full-scale drinking water supply system. Water Res. 2018; 135:11-21.
675 20.	Liu G, Zhang Y, van der Mark E, Magic-Knezev A, Pinto A, van den Bogert B, Liu
676 W, van der Meer W, Medema G. Assessing the origin of bacteria in tap water and
677 distribution system in an unchlorinated drinking water system by SourceTracker using
678 microbial community fingerprints. Water Res. 2018; 138: 86-96.
679 21.	Dias CFV, Durand A-A, Constant P, Prévost M, Bédard E. Identification of factors
680 affecting bacterial abundance and community structures in a full-scale chlorinated
681 drinking water distribution system. Water. 2019; 11. DOI: 10.3390/w11030627.
682 22.	Ling F, Whitaker R, LeChevallier MW, Liu W-T. Drinking water microbiome
683 assembly induced by water stagnation. ISME J. 2018; 12: 1520-1531.
684 23.	Lautenschlager K, Boon N, Wang Y, Egli T, Hammes F. Overnight stagnation of
685 drinking water in household taps induces microbial growth and changes in community
686 composition. Water Res, 2010; 44: 4868-4877.
687 24.	Prest EI, Hammes F, Kötzsch S, van Loosdrecht MCM, Vrouwenvelder JS.
688 Monitoring microbiological changes in drinking water systems using a fast and
689 reproducible flow cytometric method. Water Res. 2013; 47: 7131-7142.
690 25.	van der Wielen PWJJ, Brouwer-Hanzens A, Italiaander R, Hijnen WAM. Initiating
691 guidance values for novel biological stability parameters in drinking water to control
692 regrowth in the distribution system. Sci Total Environ. 2023; 871: 161930.
693 26.	Vavourakis CD, Heijnen L, Peters MCFM, Marang L, Ketelaars HAM, Hijnen WAM.
694 Spatial and temporal dynamics in attached and suspended bacterial communities in
695 three drinking water distribution systems with variable biological stability. Environ
696 Sci Technol. 2020; 54: 14535-14546.
697 27.	(OECD) OfEC-OaD. Water governance in the Netherlands: Fit for the future, abstr
698 OECD, Paris, France. 2014.
699 28.	Vohland K, Land-Zandstra A, Ceccaroni L, Lemmens R, Perelló JMM, Ponti M,
700 Samson R, Wagenknecht K. The science of citizen science. Springer Cham. 2021.
701 29.	Farnham DJ, Gibson RA, Hsueh DY, McGillis WR, Culligan PJ, Zain N, Buchanan R.
702 Citizen science-based water quality monitoring: Constructing a large database to
703 characterize the impacts of combined sewer overflow in New York City. Sci Total
704 Environ. 2017; 580: 168-177.
705 30.	Hardison DR, Holland WC, Currier RD, Kirkpatrick B, Stumpf R, Fanara T, Burris D,
706 Reich A, Kirkpatrick GJ, Litaker RW. HABscope: A tool for use by citizen scientists
707 to facilitate early warning of respiratory irritation caused by toxic blooms of Karenia
708 brevis. PLoS One. 2019; 14: e0218489.
709 31.	Gebert MJ, Delgado-Baquerizo M, Oliverio AM, Webster TM, Nichols LM, Honda
710 JR, Chan ED, Adjemian J, Dunn RR, Fierer N. Ecological analyses of mycobacteria in
711 showerhead biofilms and their relevance to human health. mBio. 2018; 9: e01614-18.
712 32.	D'Alessio M, Rushing G, Gray TL. Monitoring water quality through citizen science
713 while teaching STEM undergraduate courses during a global pandemic. Sci Total
714 Environ. 2021; 779: 146547.
715 33.	Neu HM, Lee M, Pritts JD, Sherman AR, Michel SLJ. Seeing the “Unseeable,” A
716 student-led activity to identify metals in drinking water. J Chem Educ. 2020; 97:
717 3690-3696.
718 34.	Olson CA, Malakar A, D’Alessio M, Schumacher N, Ray C, Snow DD. Evaluating
719 youth-led citizen science for improved monitoring of domestic well water quality in
720 Nebraska. ACS ES&T Water. 2024; 4: 601-609.
721 35.	Redmon JH, Levine KE, Aceituno AM, Litzenberger K, Gibson JM. Lead in drinking
722 water at North Carolina childcare centers: Piloting a citizen science-based testing
723 strategy. Environ Res. 2020; 183: 109126.
724 36.	Peckenham JM, Thornton T, Peckenham P. Validation of student generated data for
725 assessment of groundwater quality. J Sci Educ Technol. 2012; 21: 287-294.
726 37.	Brouwer S, van der Wielen PWJJ, Schriks M, Claassen M, Frijns J. Public
727 participation in science: The future and value of citizen science in the drinking water
728 research. Water (Switzerland). 2018; 10: 284.
729 38.	Hijnen WAM, Schurer R, Bahlman JA, Ketelaars HAM, Italiaander R, van der Wal A,
730 van der Wielen PWJJ. Slowly biodegradable organic compounds impact the
731 biostability of non-chlorinated drinking water produced from surface water. Water
732 Res. 2018; 129: 240-251.
733 39.	van der Kooij D, Martijn B, Schaap PG, Hoogenboezem W, Veenendaal HR, van der
734 Wielen PWJJ. Improved biostability assessment of drinking water with a suite of test
735 methods at a water supply treating eutrophic lake water. Water Res. 2015; 87: 347-
736 355.
737 40.	van der Wielen PWJJ, van der Kooij D. Nontuberculous mycobacteria, fungi, and
738 opportunistic pathogens in unchlorinated drinking water in the Netherlands. Appl
739 Environ Microbiol. 2013; 79: 825-834.
740 41.	Le Dantec C, Duguet JP, Montiel A, Dumoutier N, Dubrou S, Vincent V. Occurrence
741 of mycobacteria in water treatment lines and in water distribution systems. Appl
742 Environ Microbiol. 2002; 68: 5318-5325.
743 42.	Zhang C, Struewing I, Mistry JH, Wahman DG, Pressman J, Lu J. *Legionella* and
744 other opportunistic pathogens in full-scale chloraminated municipal drinking water
745 distribution systems. Water Res. 2021; 205: 117571.
746 43.	LeChevallier MW, Welch NJ, Smith DB. Full-scale studies of factors related to
747 coliform regrowth in drinking water. Appl Environ Microbiol. 1996; 62: 2201-2211.
748 44.	van der Kooij D, Veenendaal HR, van der Mark EJ, Dignum M. Assessment of the
749 microbial growth potential of slow sand filtrate with the biomass production potential
750 test in comparison with the assimilable organic carbon method. Water Res. 2017; 125:
751 270-279.
752 45.	Pluym T, Waegenaar F, De Gusseme B, Boon N. Microbial drinking water monitoring
753 now and in the future. Microb Biotechnol. 2024; 17: e14532.
754 46.	Safford HR, Bischel HN. Flow cytometry applications in water treatment, distribution,
755 and reuse: A review. Water Res. 2019; 151: 110-133.
756 47.	Abkar L, Moghaddam HS, Fowler SJ. Microbial ecology of drinking water from
757 source to tap. Sci Total Environ. 2024; 908: 168077.
758 48.	Bédard E, Laferrière C, Déziel E, Prévost M. Impact of stagnation and sampling
759 volume on water microbial quality monitoring in large buildings. PLOS one. 2018; 13:
760 e0199429.
761 49.	Feazel LM, Baumgartner LK, Peterson KL, Frank DN, Harris JK, Pace NR.
762 Opportunistic pathogens enriched in showerhead biofilms. Proc Natl Acad Sci USA.
763 2009; 106: 16393-16399.
764 50.	Inkinen J, Kaunisto T, Pursiainen A, Miettinen IT, Kusnetsov J, Riihinen K,
765 Keinänen-Toivola MM. Drinking water quality and formation of biofilms in an office
766 building during its first year of operation, a full scale study. Water Res. 2014; 49: 83-
767 91.
768 51.	Li J, Ren A, van der Mark E, Liu G. Direct evidence of microbiological water quality
769 changes on bacterial quantity and community caused by plumbing system. J Environ
770 Sci. 2022; 116: 175-183.
771 52.	Proctor CR, Rhoads WJ, Keane T, Salehi M, Hamilton K, Pieper KJ, Cwiertny DM,
772 Prévost M, Whelton AJ. Considerations for large building water quality after extended
773 stagnation. AWWA Water Sci. 2020; 2: e1186.
774 53.	Vincent M, Hartemann P, Engels-Deutsch M. Antimicrobial applications of copper.
775 Int J Hyg Environ Health. 2016; 219: 585-591.
776 54.	van der Kooij D, Oranje JP, Hijnen WA. Growth of *Pseudomonas aeruginosa* in tap
777 water in relation to utilization of substrates at concentrations of a few micrograms per
778 liter. Appl Environ Microbiol. 1982; 44: 1086-1095.
779 55.	Dweh TJ, Nayak J, Rout P. Food storage and preservation. 2024. pp 102-115.

780	DOI:10.58532/V3BCAG24CH9.

781 56.	Fung DYC. Food spoilage, preservation and quality control. *In* Schaechter M (ed),
782 Encyclopedia of Microbiology (Third Edition). Academic Press, Oxford. 2009; pp. 54-
783 79. DOI:https://doi.org/10.1016/B978-012373944-5.00122-X.
784 57.	Caporaso JG, Lauber CL, Walters WA, Berg-Lyons D, Lozupone CA, Turnbaugh PJ,
785 Fierer N, Knight R. Global patterns of 16S rRNA diversity at a depth of millions of
786 sequences per sample. Proc Natl Acad Sci USA. 2011; 108: 4516.

787

788 *Supporting information*
789 *Fig S1. Map of Amsterdam with the locations of the citizen scientists participating in the study*
790 *marked with blue circles.*
791 *Fig S2. Total cell numbers in drinking water sampled at 43 locations in the distribution*
792 *system of treatment Plant A (blue symbols), Plant B (yellow symbols), mixed zone of Plant A*
793 *and B (red symbols) and in treated water sampled at plant A or B (green symbols).*
794 *Fig S3. Membrane-intact cell numbers in drinking water sampled at 43 locations in the*
795 *distribution system of treatment Plant A (blue symbols), Plant B (yellow symbols), mixed zone*
796 *of Plant A and B (red symbols) and in treated water sampled at plant A or B (green symbols).*
797 *Table S1. Number of sequences, OTUs and the Shannon diversity index for each sample.*
798 *Table S2. Taxonomic classification of OTUs with ≥ 10% dominance in the community of*
799 *flushed, direct or stored drinking water samples as well as their relative abundance in the*
800 *other water types at the same location.*

801

802

Figure 1	[Click here to access/download;Figure;Fig 1.tif](https://www2.cloud.editorialmanager.com/pone/download.aspx?id=40132473&guid=74d833c6-a102-4a9f-80e2-a89bcca673da&scheme=1)[<https://www2.cloud.editorialmanager.com/pone/download.aspx?id=40132473&guid=74d833c6-a102-4a9f-80e2-a89bcca673da&scheme=1>](https://www2.cloud.editorialmanager.com/pone/download.aspx?id=40132473&guid=74d833c6-a102-4a9f-80e2-a89bcca673da&scheme=1)

Figure 2	[Click here to access/download;Figure;Fig 2.tif](https://www2.cloud.editorialmanager.com/pone/download.aspx?id=40132474&guid=6c22c685-6f30-459a-8f25-b251bb996abc&scheme=1)[<https://www2.cloud.editorialmanager.com/pone/download.aspx?id=40132474&guid=6c22c685-6f30-459a-8f25-b251bb996abc&scheme=1>](https://www2.cloud.editorialmanager.com/pone/download.aspx?id=40132474&guid=6c22c685-6f30-459a-8f25-b251bb996abc&scheme=1)

Figure 3	[Click here to access/download;Figure;Fig 3.tif](https://www2.cloud.editorialmanager.com/pone/download.aspx?id=40132476&guid=881d173b-d17d-4a3a-9908-4256116c55ee&scheme=1)[<https://www2.cloud.editorialmanager.com/pone/download.aspx?id=40132476&guid=881d173b-d17d-4a3a-9908-4256116c55ee&scheme=1>](https://www2.cloud.editorialmanager.com/pone/download.aspx?id=40132476&guid=881d173b-d17d-4a3a-9908-4256116c55ee&scheme=1)

Figure 4	[Click here to access/download;Figure;Fig 4.tif](https://www2.cloud.editorialmanager.com/pone/download.aspx?id=40132478&guid=da472f97-93e7-4f53-9ad3-5996266fac25&scheme=1)[<https://www2.cloud.editorialmanager.com/pone/download.aspx?id=40132478&guid=da472f97-93e7-4f53-9ad3-5996266fac25&scheme=1>](https://www2.cloud.editorialmanager.com/pone/download.aspx?id=40132478&guid=da472f97-93e7-4f53-9ad3-5996266fac25&scheme=1)

Supporting Information

## [Click here to access/download](https://www2.cloud.editorialmanager.com/pone/download.aspx?id=40132405&guid=a0da58c5-0185-4b61-b03b-b805215802b9&scheme=1)

[**Supporting Information** Supporting information.docx](https://www2.cloud.editorialmanager.com/pone/download.aspx?id=40132405&guid=a0da58c5-0185-4b61-b03b-b805215802b9&scheme=1)
